# Sishen wan modulates gut microbial and short-chain fatty-acid imbalances and ameliorates behavioral and inflammatory abnormalities in mice with chronic sleep deprivation

**DOI:** 10.3389/fmicb.2026.1944977

**Published:** 2026-09-03

**Authors:** Yu Jiao, Yichun Zhao, Zhongqiang Zhang, Hui Sun, Yaoai Wang, Xueke Bai, Chengyu Piao, Xia Lei, Ning Zhang, Hongdan Xu

**Affiliations:** 1 College of Pharmacy, Heilongjiang University of Chinese Medicine, Harbin, Heilongjiang, China; 2 Heilongjiang Provincial Key Laboratory of Precise Diagnosis and Neuropsychological Regulation of Mental Disorders, Qiqihar, Heilongjiang, China; 3 Wuxi Affiliated Hospital of Nanjing University of Chinese Medicine, Nanjing University of Chinese Medicine, Wuxi, Jiangsu, China; 4 Jiangsu CM Clinical Innovation Center of Degenerative Bone & Joint Disease, Wuxi, Jiangsu, China; 5 Department of Pharmacy, Wuxi Health Higher Vocational and Technical School, Wuxi, Jiangsu, China

**Keywords:** chronic sleep deprivation, fecal microbiota transplantation, gut microbiota, inflammation, short-chain fatty acids, Sishen wan

## Abstract

**Background:**

Chronic sleep deprivation (CSD) disrupts mood-related behavior, gut microbial ecology, and inflammatory homeostasis. Sishen Wan (SSW), a medicinal plant formula used clinically for chronic diarrhea, has shown microbiota- and inflammation-modulating effects in colitis models, but its protective effects under sleep-deprivation conditions remain unclear.

**Methods:**

The chemical profile of SSW was characterized by ultra-performance liquid chromatography–quadrupole time-of-flight mass spectrometry. Male C57BL/6 J mice underwent CSD using a modified multiple-platform method and received three doses of SSW or fluoxetine. Behavioral performance, histopathology, gut microbiota composition, and short-chain fatty acid (SCFA) concentrations in feces, serum, and hippocampal tissue were assessed. Fecal material from Control, Model, and high-dose SSW (SSW-H) donors was transplanted into antibiotic-pretreated recipients. Serum untargeted metabolomics, hippocampal transcriptomics, RT-qPCR, and resting-state functional magnetic resonance imaging were used to characterize metabolic, transcriptional, and brain-function changes.

**Results:**

CSD reduced sucrose preference and open-field activity, prolonged immobility in the tail-suspension and forced-swim tests, aggravated colonic and hippocampal injury, and increased pro-inflammatory cytokines. SSW-H produced the most consistent improvements. CSD also reduced gut microbial richness and diversity, altered community composition, and lowered fecal acetate, propionate, and butyrate and serum acetate. SSW-H shifted the community toward the Control profile, increased Akkermansia, reduced several Model-enriched taxa, and increased major fecal SCFAs and serum acetate. Recipients of Model-donor feces developed reduced sucrose preference and activity, prolonged immobility, and colonic and hippocampal abnormalities. In contrast, recipients of SSW-H-donor feces showed milder behavioral and histological changes and a microbial profile distinct from that of FMT-Model recipients. SSW-H was also associated with partial normalization of lipid-, amino-acid-, and one-carbon-metabolism-related serum features, modulation of hippocampal immune pathways involving chemokines, cytokines, and NF-κB signaling, and attenuation of several CSD-associated regional brain abnormalities.

**Conclusion:**

SSW alleviated CSD-associated behavioral abnormalities, tissue injury, and inflammation, with the high dose showing the most consistent effects. These improvements were accompanied by changes in gut microbial composition and SCFA profiles.

## 1 Introduction

Insufficient sleep and persistent sleep disturbances constitute a substantial global public-health problem. A systematic review conducted in 2025 estimated that approximately 16.2% of adults worldwide, representing more than 850 million people, met the criteria for insomnia disorder, although insomnia disorder is only one clinical manifestation of sustained sleep impairment (Benjafield et al., 2025). Habitually short sleep durations are associated with a higher risk of psychiatric conditions, including anxiety and depression, as well as obesity, impaired glucose metabolism, cardiovascular disease, and all-cause mortality (Zhang J. et al., 2024; Itani et al., 2017). As sleep loss accumulates, its consequences extend beyond circadian disruption to neuroendocrine, immune, metabolic, and gastrointestinal homeostasis. Experimental studies have also shown that sleep deprivation weakens neuronal connectivity in hippocampal area CA1 and promotes glial activation, indicating concurrent effects on the neural circuits and the cerebral immune environment (Havekes et al., 2016; Bellesi et al., 2017). Characterizing the development of these multisystem abnormalities and identifying interventions capable of addressing both peripheral and central changes remain important objectives in sleep-related research.

Sleep loss can persistently disturb the gut microbial ecosystem while affecting central nervous system function, raising the possibility that microbial alterations amplify systemic responses to disrupted sleep. Chronic sleep fragmentation and repeated sleep disturbances alter gut microbial composition and metabolism and are accompanied by impaired intestinal barrier integrity, systemic inflammation, and metabolic dysfunction (Poroyko et al., 2016; Bowers et al., 2020). Yang et al. reported that acute sleep deprivation simultaneously disrupted circadian rhythms, the gut microbiota, and systemic inflammatory status (Yang et al., 2023), whereas recurrent partial sleep deprivation in humans was associated with changes in the microbial composition and glucose metabolism (Benedict et al., 2016). The microbial fluctuations occurring after brief sleep deprivation are generally modest, but longer exposure tends to produce more pronounced shifts in community structure and metabolic function (El Aidy et al., 2020; Li L. et al., 2024). Sleep and the gut microbiota also exhibit marked temporal rhythmicity (Shan et al., 2025; Matenchuk et al., 2020). Although the specific taxa reported across studies are not fully consistent, the broader evidence supports sustained sleep loss as a disruptor of gut microbial structure and function. More importantly, microbiota depletion, reconstruction, or transplantation can alter the inflammatory responses, barrier injury, and neurobehavioral abnormalities after sleep deprivation, indicating that dysbiosis is not merely an accompanying observation (Wang et al., 2021; Zhao et al., 2023; Wang et al., 2025). Therefore, studies of CSD-associated injury should consider whether the microbial changes are followed by functional consequences that can be sensed by the host.

Many functional effects of the gut microbiota are conveyed by microbial metabolites, of which short-chain fatty acids (SCFAs)—particularly acetic acid, propionic acid, and butyric acid—have been studied most extensively. These metabolites are mainly produced through bacterial fermentation of nondigestible carbohydrates and contribute to colonocyte energy supply, mucosal barrier maintenance, and immune homeostasis. They can also affect distant tissues through free–fatty-acid receptors, histone deacetylase inhibition, and neurohumoral routes (Cryan et al., 2019; Dalile et al., 2019; Smith et al., 2013). Restoration of microbiota-derived metabolites such as butyric acid has been shown to promote sleep and reduce the intestinal mucosal injury caused by insufficient sleep (Szentirmai et al., 2019; Gao et al., 2023; Wang X. et al., 2023). Studies by Gao et al. further linked the protective effects of melatonin against sleep-deprivation-induced barrier dysfunction and colitis to changes in the gut microbiota and butyric acid (Gao et al., 2019; Gao et al., 2021). In humans, fecal SCFA concentrations have been shown to correlate with sleep continuity in older adults with insomnia symptoms, and differences in sleep quality are accompanied by alterations in microbial metabolic pathways (Magzal et al., 2021; Seong et al., 2024). The gut microbiota and its metabolic environment can also influence blood–brain barrier integrity and microglial homeostasis, providing a biological basis for communication between peripheral microbial changes and cerebral responses (Braniste et al., 2014; Erny et al., 2015).

After oral administration of traditional medicinal formulas, poorly absorbed or structurally complex constituents may remain in the intestine for extended periods and undergo microbial hydrolysis, reduction, and other biotransformations that alter their chemical forms and biological activities (Zhang et al., 2023; Liu et al., 2019). This concept has driven renewed evaluation of medicinal formulas from the microbiome and metabolite perspectives. In models of mood disturbance, the Jia Wei Xiao Yao San and Gan-Mai-Da-Zao decoctions have been reported to alter gut microbial composition, with behavioral improvements occurring alongside changes in the levels of microbial metabolites and inflammatory markers (Ji et al., 2022; Zhao et al., 2025; Zhang B. et al., 2025). Similarly, an interventional study in patients with insomnia showed that improved sleep after treatment with traditional Chinese herbal formulas was accompanied by changes in the microbial structure and inflammatory indices (Zeng et al., 2024). Suanzaoren decoction produced different microbial and metabolic responses in insomnia and depression models, indicating that the microbiological effects of the same formula may depend on the pathological context (Zhang H. et al., 2024). In sleep-deprived mice, the improvements in cognitive impairment induced by chlorogenic acid were accompanied by changes in the gut microbiota and immune function (Zhu et al., 2024). Studies of Zhi-zi-chi decoction and Jiaotaiwan have provided additional evidence linking the mood-related effects of medicinal formulas to microbial metabolism and SCFA-associated signaling, respectively (He et al., 2025; Huang et al., 2025). Beyond classical formulas, polysaccharides and oligosaccharides derived from individual medicinal plants have also shown intestinal protective effects associated with the microbiota and SCFAs. *Artemisia argyi* polysaccharides were shown to reduce intestinal inflammation and correct dysbiosis in lipopolysaccharide-treated mice (Ning et al., 2024), whereas *Dendrobium officinale* oligosaccharides protected against chronic colitis in association with changes in inflammation and the gut microbiota (Shi et al., 2025). Collectively, these studies identified microbial composition and metabolites as important dimensions of medicinal-formula pharmacology; however, most of these studies focused on colitis or conventional mood-disorder models rather than the multisystem abnormalities associated with sleep deprivation.

Sishen Wan (SSW), which is composed of *Psoralea corylifolia*, *Myristica fragrans*, *Euodia rutaecarpa*, and *Schisandra chinensis*, is used clinically for chronic diarrhea and intestinal dysfunction. In traditional Chinese medicine, SSW is commonly used for chronic diarrhea associated with spleen–kidney yang deficiency. In experimental colitis, SSW reduces colonic injury and inflammatory responses, and these effects are accompanied by changes in gut microbial composition, fecal metabolites, Toll-like receptor 4 (TLR4)/nuclear factor (NF)-κB signaling, and the Treg/Th17 balance. Among these studies, Ge et al. used a colitis model with spleen–kidney yang deficiency and reported concurrent changes in gut microbial composition, fecal metabolites, and inflammatory responses after SSW treatment (Wang et al., 2019; Chen et al., 2020; Ge et al., 2022; Wang et al., 2022). In addition, a clinical study of insomnia reported differences in gut microbial profiles among traditional Chinese medicine syndrome types and treatment-associated microbial changes after Chinese herbal therapy. These observations provide a traditional and microbiological rationale for examining SSW in a sleep-deprivation model with concurrent intestinal and neurobehavioral abnormalities, although the present CSD model was not designed to represent spleen–kidney yang deficiency. However, most previous evidence for the effects of SSW on the intestinal microenvironment has been obtained in models of localized colonic disease. Because CSD is associated with mood-related behavioral abnormalities, gut microbial dysbiosis, and SCFA depletion, we hypothesized that SSW may protect against CSD-associated injury in association with an improved gut microbial state. Therefore, we first examined the effects of SSW on behavior, gut microbial composition, SCFAs, and colonic and hippocampal inflammation in CSD mice. We then performed fecal microbiota transplantation (FMT) by using fecal material from Control, Model, and SSW-H donors in antibiotic-pretreated recipients to determine whether the distinct donor-associated intestinal states were accompanied by different behavioral and pathological phenotypes after transplantation. Serum untargeted metabolomics, hippocampal transcriptomics, and resting-state functional magnetic resonance imaging (rs-fMRI) were further used to characterize the metabolic, molecular, and brain-function changes associated with SSW treatment.

## 2 Materials and methods

### 2.1 Animals and ethical approval

A total of 72 eight-week-old male C57BL/6 J mice were used in the direct-intervention experiment, and 18 additional male C57BL/6 J mice were used as FMT recipients. The animals were purchased from Liaoning Changsheng Biotechnology Co., Ltd. and maintained under specific pathogen-free conditions at 23 °C ± 1 °C and 55% ± 5% relative humidity with a 12-h light/12-h dark cycle and ad libitum access to food and water. The experiments began after a 1-week acclimation period. All procedures were approved by the Animal Ethics Committee of Heilongjiang University of Chinese Medicine (Approval No. 2025050935) and were conducted in accordance with the applicable animal-welfare and reporting guidelines.

### 2.2 SSW sourcing, chemical characterization, dosing, and group allocation

SSW was purchased from Beijing Tongrentang Natural Medicine (Tangshan) Co., Ltd. (batch No. 23080014). To characterize the phytochemical background of the intervention, the batch used in this study was analyzed by ultra-performance liquid chromatography–quadrupole time-of-flight mass spectrometry (UPLC–QTOF–MS) in positive- and negative-ion modes. Candidate constituents were putatively annotated on the basis of retention time, accurate mass, adduct form, and tandem mass spectrometry (MS/MS) fragmentation. After combining data from both ionization modes and removing duplicate annotations, 92 putative constituents were retained. Representative total-ion chromatograms are shown in Supplementary Figure S1, and the complete annotation is provided in Supplementary Methods S1 and Supplementary Table S1. Before each administration, SSW was suspended in purified water. Doses were converted from the adult clinical dose on the basis of body-surface area and were set at 1.25, 2.5, and 5.0 g/kg for the SSW-L, SSW-M, and SSW-H groups, respectively. Fluoxetine (FLX; 10 mg/kg) was used as a positive behavioral comparator. All treatments were administered by oral gavage at a fixed time each day. After acclimation and before the start of any intervention, the 72 mice were allocated to the Control, Model, FLX, SSW-L, SSW-M, and SSW-H groups (*n* = 12 per group) by simple randomization. No separate formal allocation-concealment procedure was implemented. Behavioral video analysis and histological evaluation were subsequently performed by investigators blinded to group allocation.

### 2.3 CSD induction and drug treatment

CSD was induced using a modified multiple-platform water-environment method (Machado et al., 2004). Except for the mice in the Control group, all mice were placed in tanks containing multiple small platforms, with the water level maintained approximately 2 cm below the platform surface. Food and water were provided on a rack above the tank. Sleep deprivation was imposed from 16:00 to 10:00 the following day for 21 consecutive days. When the animal’s muscle tone declined during sleep and the animal came into contact with the water, it was awakened, thereby interrupting sustained sleep. Control mice did not undergo water-environment sleep deprivation; instead, their bedding was moistened for the same duration. Beginning on day 1, the mice in the Control and Model groups received an equivalent volume of purified water by gavage, whereas those in the FLX and SSW groups received their assigned treatments. Behavioral testing and rs-fMRI acquisition were completed during the final 5 days of the CSD protocol.

### 2.4 Behavioral tests

Behavioral testing consisted of the open-field test (OFT), sucrose-preference test (SPT), tail-suspension test (TST), and forced-swim test (FST). For the OFT, each mouse was placed in the center of the arena and allowed to explore freely for 5 min. The total distance traveled and distance traveled in the central zone were recorded. Before the SPT, the mice were habituated to 1% sucrose solution and then deprived of food and water for 12 h. They were subsequently provided with one bottle of 1% sucrose solution and one bottle of purified water; the bottle positions were counterbalanced. Fluid intake was recorded after 12 h, and sucrose preference was calculated as sucrose intake/[sucrose intake + water intake] × 100%. The TST lasted 6 min, and immobility during the final 5 min was quantified. For the FST, the mice were placed in water maintained at 24 °C-26 °C for 6 min; the first minute served as the habituation period, and immobility during the final 5 min was recorded. Immobility in the TST was defined as cessation of active struggling, whereas immobility in the FST was defined by the presence of only the minimal movements required to keep the head above water. The apparatus was cleaned with 75% ethanol after each animal. Video acquisition, trajectory analysis, and behavioral scoring were performed by investigators blinded to group allocation. Motor coordination and endurance were additionally evaluated using the rotarod and treadmill tests. For the rotarod test, the rotation speed increased from 4 to 40 rpm over 5 min. Each mouse completed three trials at 15 min intervals, and the mean latency to fall was used for analysis. For the treadmill endurance test, mice were acclimated to treadmill running before testing. The speed was increased from 5 to 21 m/min using a graded protocol, and running continued until exhaustion. The time to exhaustion was recorded.

### 2.5 Resting-state functional magnetic resonance imaging

Mice in the Control, Model, and SSW-H groups underwent 9.4 T magnetic resonance imaging at Harbin Institute of Technology, China. Anesthesia was induced with 3% isoflurane and maintained at approximately 2%. The head was stabilized with ear bars and a bite bar, and respiratory rate was monitored continuously during acquisition. Structural images were acquired using a rapid acquisition with relaxation enhancement (RARE) sequence with the following parameters: axial slices, 30; repetition time (TR), 5,000 ms; echo time (TE), 36 ms; slice thickness, 0.7 mm; no interslice gap; matrix, 256 × 256; field of view (FOV), 20 × 20 mm; and flip angle, 180°. Functional images were acquired using an echo-planar imaging sequence with the following parameters: axial slices, 25; TR, 2,000 ms; TE, 15 ms; slice thickness, 0.4 mm; no interslice gap; matrix, 64 × 64; FOV, 20 × 20 mm; and flip angle, 90°. Images were processed using DPABI and SPMAnimalIHEP. Preprocessing included format conversion, resampling, slice-timing correction, motion correction, tissue segmentation, spatial normalization, smoothing, and denoising. Structural images were evaluated by voxel-based morphometry (VBM), and the amplitudes of low-frequency fluctuations (ALFFs) and regional homogeneity (ReHo) were calculated from the functional data. Voxel-wise pairwise comparisons were performed using two-sample t tests with a voxel threshold of *p* < 0.05 and a cluster size > 20 voxels.

### 2.6 Sample collection, tissue processing, and histopathology

After the final treatment, mice were placed individually in clean cages lined with sterile filter paper. Fresh fecal samples were collected after gentle stimulation of the lower abdomen, snap-frozen in liquid nitrogen, and stored at −80 °C for 16S rRNA gene sequencing and preparation of FMT donor material. Blood was collected from the retro-orbital sinus under isoflurane inhalation anesthesia induced at 3% and maintained at approximately 2%. After blood collection, the mice were maintained under deep isoflurane anesthesia, as confirmed by the absence of the pedal withdrawal reflex. Mice used for fresh-tissue analyses were euthanized by cervical dislocation, and death was confirmed by the absence of spontaneous respiration and heartbeat before tissue collection. Mice designated for histopathological examination were transcardially perfused with physiological saline under deep anesthesia, after which cervical dislocation was performed to ensure death before tissue collection. Blood samples were allowed to stand at 4 °C for 30 min and centrifuged at 3,500 r/min for 10 min at 4 °C. Serum and fresh tissue samples were stored at −80 °C until analysis. Colon and brain tissues collected for histopathology were fixed in 4% paraformaldehyde, dehydrated through graded ethanol, cleared in xylene, embedded in paraffin, and sectioned. H&E staining was used to evaluate colonic histopathology and neuronal morphology in the hippocampal CA1 and CA3 regions. Nissl staining was used to assess hippocampal neuronal morphology and Nissl bodies. Image acquisition and histological evaluation were performed by investigators blinded to group allocation.

### 2.7 Cytokine measurements

Colon and hippocampal tissues were thoroughly homogenized in precooled homogenization buffer on ice, and the supernatants were collected after centrifugation. Serum samples were thawed on ice and clarified by centrifugation before analysis. Mouse interleukin (IL)-1β, IL-6, tumor necrosis factor (TNF)-α, and IL-10 were measured using commercial enzyme-linked immunosorbent assay kits purchased from Nanjing Jiancheng Bioengineering Institute Co., Ltd. (Nanjing, China), strictly in accordance with the manufacturer’s instructions. Standards and samples were analyzed in duplicate. Standard curves were generated from the absorbance values of the standards and used to calculate cytokine concentrations. Absorbance was measured at 450 nm by using a microplate reader.

### 2.8 16S rRNA gene sequencing and microbial-community analysis

For microbiome analysis, six biological replicates were used per group. After sample collection, six fecal samples were randomly selected from the available samples in each of the Control, Model, and SSW-H groups, with the same number of samples retained across groups. Sample selection was independent of behavioral, histological, cytokine, and other outcome data. The samples were snap-frozen in liquid nitrogen and stored at −80 °C. Fecal DNA was extracted using the cetyltrimethylammonium bromide method. The V3–V4 region of the 16S rRNA gene was amplified using the primers 341F (CCTAYGGGRBGCASCAG) and 806R (GGACTACNNGGGTATCTAAT), and libraries were sequenced on the NovaSeq 6,000 platform. Raw reads were quality-filtered with fastp, merged with FLASH, and screened for chimeras by using VSEARCH. Amplicon sequence variants (ASVs) were generated with Deblur in QIIME 2, followed by taxonomic annotation against the SILVA database using the Mothur method. Chao1 and Shannon indices were calculated to assess alpha diversity. Principal component analysis (PCA) was used for ordination, and between-group differences in community composition were tested by permutational multivariate analysis of variance (PERMANOVA). Linear discriminant analysis effect size (LEfSe) was performed using linear discriminant analysis (LDA) score > 4 and *p* < 0.05 as the thresholds.

### 2.9 FMT analyses

FMT was used to compare the recipient phenotypes associated with complex fecal material obtained from Control, Model, and SSW-H donors. Before preparation of the transplant suspension, fecal samples from donor mice within each group were pooled to generate a group-level donor inoculum. Recipient mice in each FMT group therefore received aliquots prepared from the corresponding pooled donor material rather than material from an individually matched donor. Frozen donor feces were thawed in a water bath at 37.5 °C for 10 min and homogenized in sterile saline at 1:10 (w/v). The suspension was vortexed for 5 min, filtered through sterile 200-mesh gauze, and centrifuged at 600 × *g* for 5 min. The resulting supernatant was used as the transplant preparation. After a 1-week acclimation period, 18 male C57BL/6 J recipient mice received an antibiotic mixture by daily oral gavage for 14 consecutive days to reduce the endogenous microbial load. The mixture contained 50 mg metronidazole, 50 mg neomycin sulfate, 50 mg ampicillin sodium, and 25 mg vancomycin in 2 mL of phosphate-buffered saline (PBS); each mouse received 200 μL of the mixture per day. Recipients were then randomly assigned to the FMT-Control, FMT-Model, and FMT-SSW groups (n = 6 per group) and received the corresponding donor material at 10 μL/g body weight three times weekly for 3 weeks. The FMT-SSW group received material from SSW-H donors. Recipient mice were not subjected to CSD and did not receive SSW or FLX directly.

### 2.10 *Akkermansia muciniphila* culture, heat inactivation, and intervention

*Akkermansia muciniphila* (ATCC BAA-835) was purchased from Mingzhou Biotechnology Co., Ltd. (Zhejiang, China) and cultured anaerobically at 37 °C. When the culture reached an OD600 of approximately 1.0, bacterial cells were collected by centrifugation at 11,000 × g for 20 min at 4 °C, washed twice with pre-reduced sterile phosphate-buffered saline (PBS), and resuspended to 2.5 × 10^9^ CFU/200 μL. Heat-inactivated *A. muciniphila* was prepared by heating the bacterial suspension at 70 °C for 30 min, and loss of cultivability was confirmed by anaerobic culture for 7 days. A total of 24 eight-week-old male C57BL/6 J Mice were randomly assigned to the Control, Model, CSD + Live Akk, and CSD + HI-Akk groups (n = 6 per group). Except for the Control group, all mice underwent CSD for 21 consecutive days as described in Section 2.3. Beginning on day 1, mice in the CSD + Live Akk and CSD + HI-Akk groups received 200 μL of live or heat-inactivated *A. muciniphila* suspension by oral gavage once daily, respectively. Control and Model mice received an equivalent volume of PBS. Behavioral testing was performed as described in Section 2.4. IL-1β, IL-6, IL-10 and TNF-α levels in colon homogenates, serum, and hippocampal homogenates were measured by ELISA as described in Section 2.7.

### 2.11 Quantification of SCFAs in feces, serum, and hippocampal tissue

Feces (100 mg), serum (100 μL), or hippocampal tissue homogenate (100 mg) was mixed with 50 μL of 20% phosphoric acid and vortexed for 2 min. Samples were then extracted with 250 μL ethyl acetate containing 4-methylpentanoic acid as the internal standard (50 μg/mL). The resulting organic supernatant was used directly for GC–MS analysis without further dilution; thus, the nominal internal-standard concentration in the final extract was 50 μg/mL. After homogenization for 1 min, the samples were centrifuged at 10,000 × *g* for 20 min at 4 °C, allowed to stand for 30 min, and the supernatant was collected for analysis. Chromatographic separation was performed on an SH-Rtx-Wax capillary column (30 m × 0.25 mm, 0.25 μm) with a 1-μL injection volume, an inlet temperature of 250 °C, and a split ratio of 10:1. Helium was used as the carrier gas at 1.0 mL/min. The oven was held at 90 °C for 2 min, increased to 200 °C at 11 °C/min, and held for 2 min. Mass spectrometry was performed with electron-impact ionization and the following parameters: ion-source temperature, 230 °C; transfer-line temperature, 240 °C; electron energy, 70 eV; and detector voltage, 1,000 V. Data were processed using Xcalibur, and analytes were quantified by the internal-standard method on the basis of the retention time and peak area. The levels of acetic acid, propionic acid, isobutyric acid, butyric acid, isovaleric acid, valeric acid, and hexanoic acid were measured in feces, while those of acetic acid, propionic acid, and butyric acid were measured in serum and hippocampal tissue. Hippocampal SCFA concentrations were normalized to tissue wet weight. Hippocampal SCFAs were quantified in the Control, Model, and SSW-H groups (*n* = 6 per group).

### 2.12 Serum untargeted metabolomics

Serum samples from the Control, Model, and SSW-H groups (*n* = 6 per group) were used for untargeted metabolomics. Serum metabolites were separated on an ACQUITY UPLC BEH C18 column (100 mm × 2.1 mm, 1.7 μm). Mobile phase A was water containing 0.1% formic acid, and mobile phase B was acetonitrile. The flow rate was 0.3 mL/min; column temperature was 40 °C; injection volume was 5 μL; and the total run time was 12 min. Data were acquired over m/z 50–1,200 in positive- and negative-ion electrospray modes. The capillary voltage was 3.0 kV in positive mode and 2.5 kV in negative mode; the cone voltage was 40 V, source temperature was 120 °C, desolvation temperature was 450 °C, cone-gas flow was 50 L/h, and desolvation-gas flow was 1,000 L/h. Data were acquired in MSE mode. Raw data were subjected to peak extraction, alignment, and normalization in Progenesis QI and then imported into MetaboAnalyst for PCA and orthogonal partial least-squares discriminant analysis (OPLS-DA). The validity of the OPLS-DA models and the potential for overfitting were further assessed using 100 permutation tests. Differential metabolic features were defined by a variable importance in projection (VIP) score > 1 and *p* < 0.05.

### 2.13 Hippocampal transcriptomics

Hippocampal transcriptomics was performed in the Control, Model, and SSW-H groups (*n* = 3 per group). Total RNA was extracted from hippocampal tissue using TRIzol. RNA quality was evaluated using an Agilent 5,300 Bioanalyzer, and concentration was measured with an ND-2000 microspectrophotometer. After quality control, libraries were prepared from 1 μg total RNA using the Illumina Stranded mRNA Prep, Ligation kit. mRNA was enriched with oligo(dT) magnetic beads and subjected to fragmentation, double-stranded complementary DNA (cDNA) synthesis, end repair, A-tailing, adapter ligation, and polymerase chain reaction (PCR) amplification. Paired-end sequencing (2 × 150 bp) was performed on the NovaSeq X Plus platform. Adapters were removed and reads were quality-filtered with fastp. Strand-specific alignment to the reference genome was performed with HISAT2; transcripts were assembled with StringTie; and expression was quantified with RNA sequencing by expectation–maximization (RSEM). Transcripts per million values were used for visualization, whereas raw counts were analyzed with DESeq2. Genes with |log2 fold change| ≥ 1 and an adjusted *p* value < 0.05 were defined as candidate differentially expressed genes (DEGs) and subjected to Gene Ontology (GO) and Kyoto Encyclopedia of Genes and Genomes (KEGG) enrichment analyses.

### 2.14 Reverse-transcription quantitative polymerase chain reaction validation

Hippocampal samples from the Control, Model, and SSW-H groups (*n* = 6 per group) were used for reverse-transcription quantitative polymerase chain reaction (RT-qPCR). RT-qPCR was used to measure the mRNA expression of *Egr2*, *Rgs4*, and *Cdkn1a*. Total hippocampal RNA was extracted with TRIzol, and genomic DNA removal and cDNA synthesis were performed using HiScript III RT SuperMix for quantitative polymerase chain reaction (qPCR; +gDNA wiper; Vazyme, China) in accordance with the manufacturer’s instructions. cDNA was diluted fivefold before amplification. Each 20-μL reaction mixture contained 10 μL of 2 × ChamQ Universal SYBR qPCR Master Mix (Vazyme, China), 0.4 μL each of forward and reverse primers (10 μM), 2 μL cDNA, and 7.2 μL nuclease-free water. Amplification was performed on a QuantStudio 5 Real-Time PCR System (Applied Biosystems, USA) with initial denaturation at 95 °C for 30 s, followed by 40 cycles of 95 °C for 10 s and 60 °C for 30 s. Melt-curve analysis was performed after amplification. *Hprt1* served as the reference gene, and relative expression was calculated using the 2^−ΔΔCt^ method. Primer sequences are provided in Supplementary Table S2.

### 2.15 Statistical analysis

Unless otherwise stated, continuous variables were presented as the mean ± standard error of the mean (SEM). Normality and homogeneity of variance were assessed before group comparisons. Data satisfying both assumptions were analyzed by one-way analysis of variance (ANOVA) followed by Tukey’s *post-hoc* test. When homogeneity of variance was not met, Welch’s ANOVA with Games–Howell post-hoc comparisons was used. Non-normally distributed data were analyzed using the Kruskal–Wallis test followed by Dunn’s *post-hoc* test. Differences in microbial-community composition were assessed by PERMANOVA. The number of biological replicates, statistical tests, post-hoc procedures, and comparisons represented by significance symbols are specified in the corresponding figure panels or legends. *p* < 0.05 was considered statistically significant.

## 3 Results

### 3.1 SSW alleviated CSD-associated behavioral abnormalities and altered exploratory neuroimaging measures

**Figure 1:**
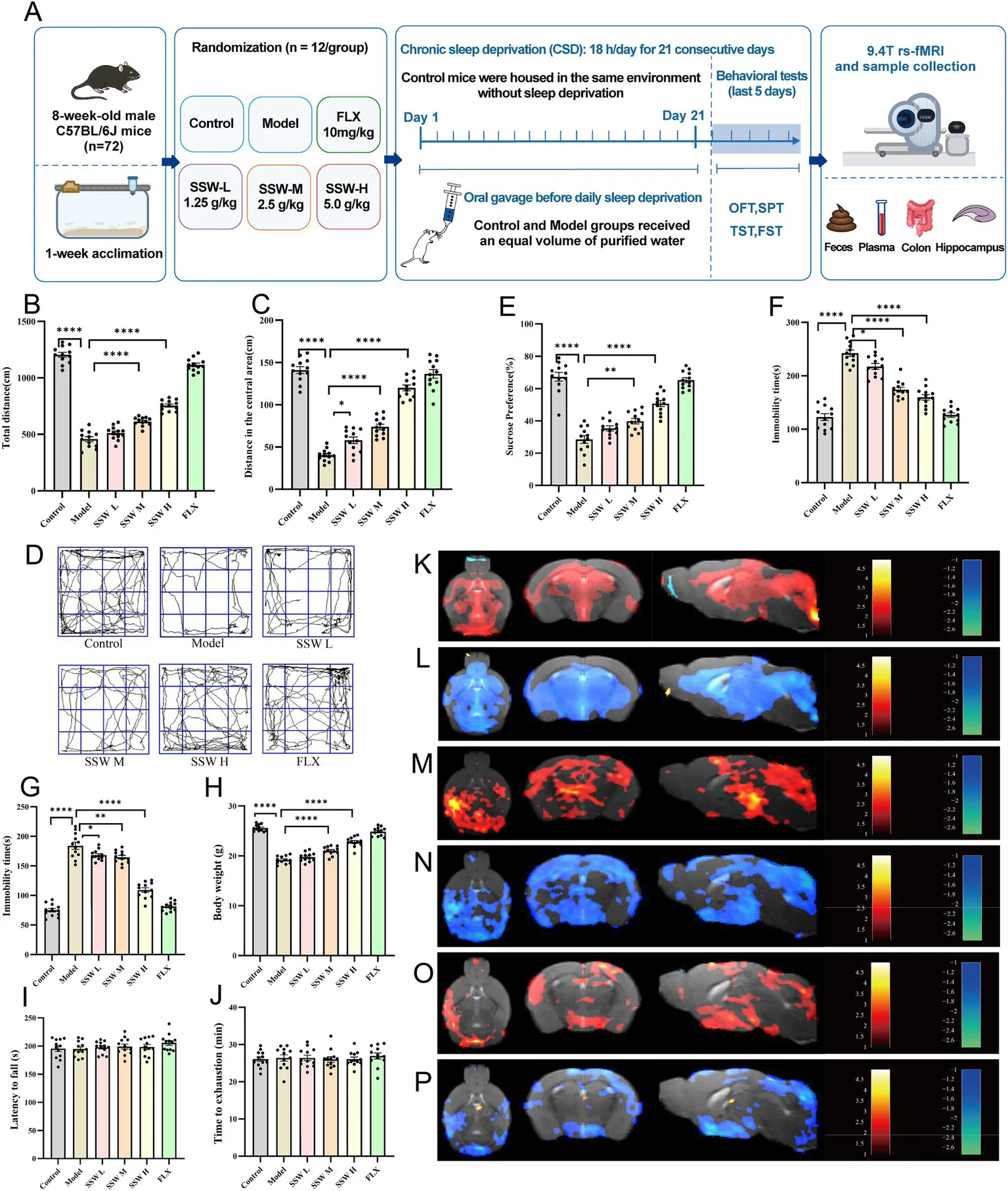
Behavioral performance, motor-function assessment, and whole-brain rs-fMRI analysis of CSD mice treated with SSW. **(A)** Experimental design. **(B,C)** Total distance traveled and distance traveled in the central zone in the OFT. **(D)** Representative OFT movement trajectories. **(E)** Sucrose preference. **(F)** TST immobility time. **(G)** FST immobility time. **(H)** Body weight. **(I)** Rotarod latency to fall. **(J)** Treadmill time to exhaustion. **(K,L)** VBM comparisons for Control versus Model and Model versus SSW-H groups, respectively. **(M,N)** ALFF comparisons for Control versus Model and Model versus SSW-H groups, respectively. **(O,P)** ReHo comparisons for Control versus Model and Model versus SSW-H groups, respectively. Behavioral, motor-function, and body-weight data are presented as mean ± SEM; each point represents one biologically independent mouse (*n* = 12 per group). MRI analysis included *n* = 6 mice per group. Voxel-wise two-sample *t*-test maps were thresholded at *p* < 0.05 with cluster size > 20 voxels. Red and blue denote higher and lower values, respectively, for the indicated comparison. Statistical procedures and significance symbols for behavioral and motor-function data are specified in the panels and Section 2.15.

The experimental design is shown in Figure 1A. In comparison with the mice in the Control group, Model mice traveled shorter total and central-zone distances in the OFT, showed lower sucrose preference, and had longer immobility times in the TST and FST (*p* < 0.05). FLX and all three doses of SSW improved subsets of these behavioral measures (*p* < 0.05), with the most consistent changes observed in the SSW-H group (Figures 1B–G). CSD also reduced body weight, whereas body weight was higher in the SSW-H group than in the Model group (Figure 1H). To assess whether the TST and FST findings could be explained by differences in general motor performance, motor coordination and endurance were additionally evaluated using the rotarod and treadmill tests. Rotarod latency to fall and treadmill time to exhaustion did not differ significantly among the six groups (*p* > 0.05; Figures 1I,J). These findings indicate that the differences in TST and FST immobility were unlikely to be explained solely by gross differences in motor coordination or endurance. Whole-brain VBM (Figures 1K,L; Supplementary Table S3), ALFF (Figures 1M,N; Supplementary Table S4), and ReHo (Figures 1O,P; Supplementary Table S5) analyses were performed in the Control, Model, and SSW-H groups. Relative to the values in the Control group, the Model group showed higher gray-matter VBM signals and higher ALFF and ReHo values in several regions, including the hippocampus and amygdala. These differences were attenuated in the SSW-H group relative to the Model group. Given the limited imaging sample size (n = 6 per group), these rs-fMRI findings were treated as exploratory and were not used as stand-alone evidence for a mechanistic conclusion.

### 3.2 SSW reduced colonic and hippocampal histopathological and inflammatory abnormalities

**Figure 2:**
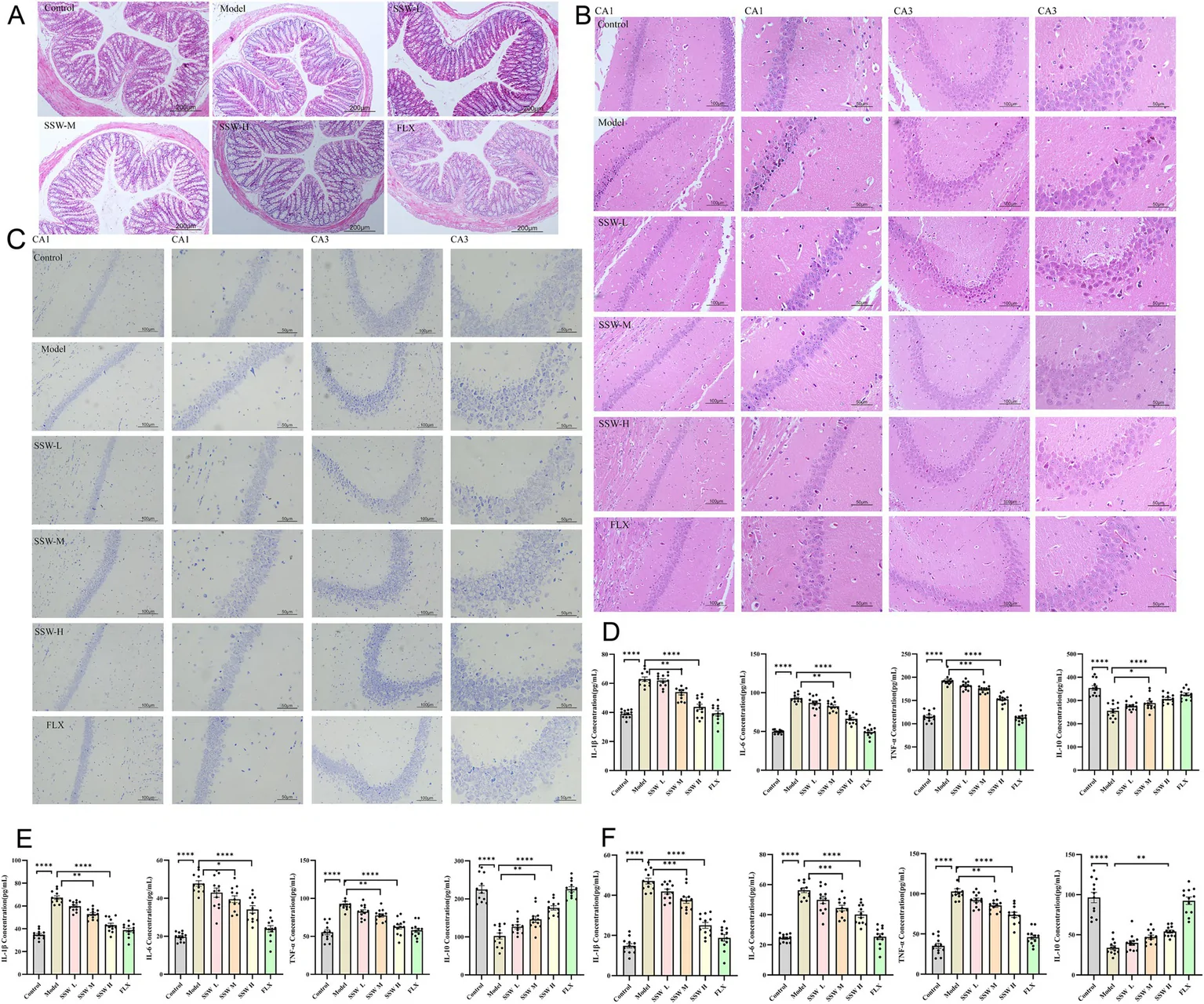
Effects of SSW on colonic and hippocampal histology and inflammatory cytokines in CSD mice. **(A)** Colonic H&E staining. **(B)** H&E staining of hippocampal CA1 and CA3. **(C)** Nissl staining of hippocampal CA1 and CA3. **(D–F)** IL-1β, IL-6, TNF-α, and IL-10 concentrations in the colon homogenate, serum, and hippocampal homogenate, respectively. The mice were categorized into Control, Model, FLX, SSW-L, SSW-M, and SSW-H groups (*n* = 12 per group). Statistical procedures and significance symbols are specified in the panels and Section 2.15.

H&E staining of the colon showed disrupted mucosal architecture, irregular crypt arrangement, fewer goblet cells, mild inflammatory-cell infiltration, and submucosal edema in the Model group in comparison with the Control group. These abnormalities were less pronounced after SSW treatment, with the clearest improvement observed in the SSW-H group (Figure 2A). Hippocampal H&E and Nissl staining showed regularly arranged neurons with intact morphology and distinct Nissl bodies in CA1 and CA3 of Control mice. Model mice showed disordered neuronal arrangement, abnormal somatic morphology, nuclear hyperchromasia or pyknosis, and fewer Nissl bodies, particularly in CA3. These changes reduced after FLX or SSW treatment (Figures 2B,C). ELISA showed that IL-1β, IL-6, and TNF-α concentrations were higher and IL-10 concentrations were lower in the colon homogenate (Figure 2D), serum (Figure 2E), and hippocampal homogenate (Figure 2F) from the Model group than from the Control group (*p* < 0.05). SSW treatment reduced the IL-1β, IL-6, and TNF-α levels and increased IL-10 levels in all three sample types (*p* < 0.05).

### 3.3 SSW modified gut microbial dysbiosis and SCFA abnormalities in CSD mice

**Figure 3:**
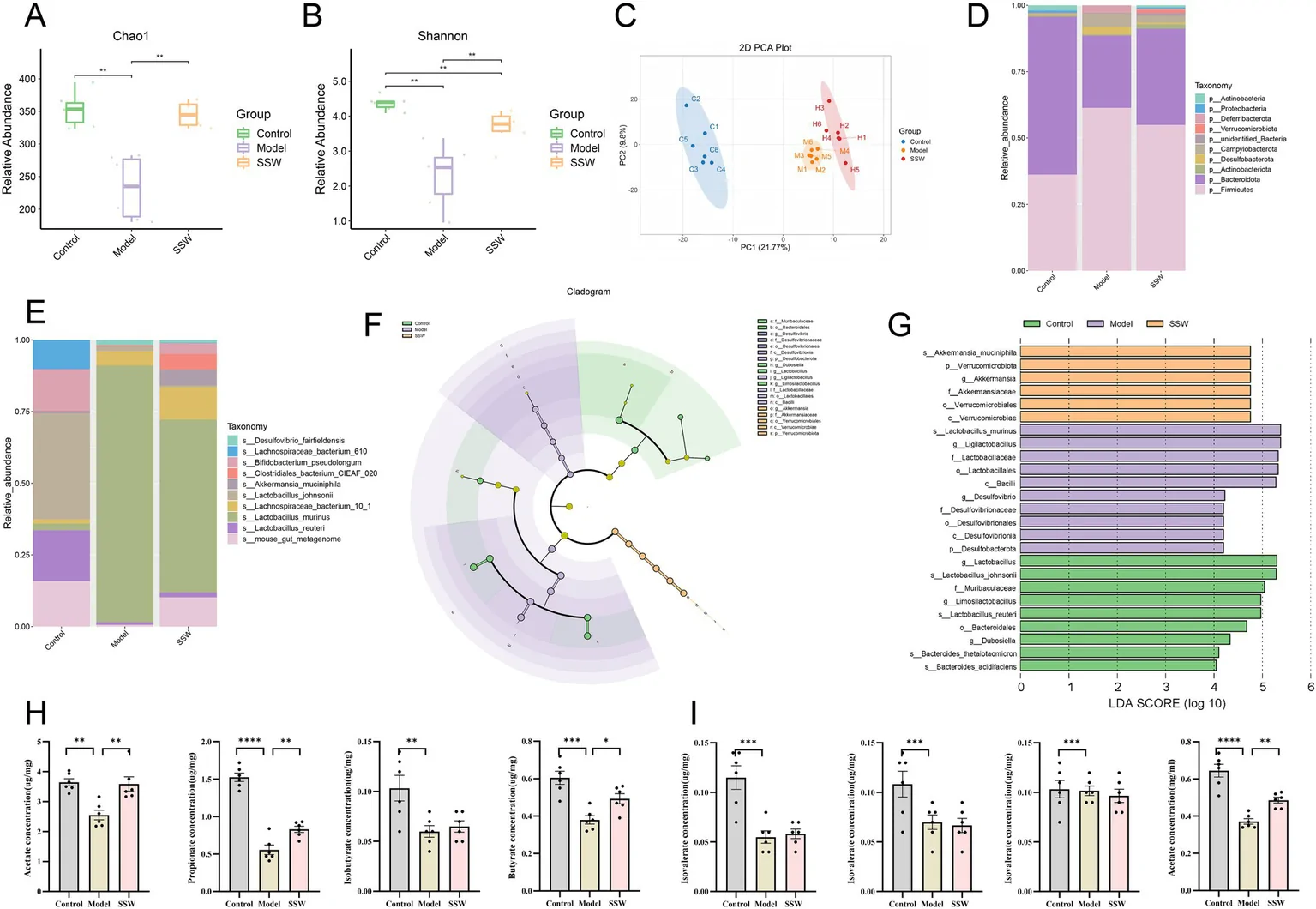
Gut microbial composition and SCFA profiles after CSD and SSW-H treatment. **(A,B)** Chao1 and Shannon indices. **(C)** PCA ordination. **(D)** Phylum-level community composition. **(E)** Species-level community composition. **(F,G)** LEfSe cladogram and LDA score plot. **(H)** Fecal SCFA concentrations. **(I)** Serum SCFA concentrations. The mice were categorized into Control, Model, and SSW-H groups (*n* = 6 per group for 16S rRNA gene sequencing). Data and significance symbols are presented in the panels; statistical procedures are described in Section 2.15.

Chao1 and Shannon indices were lower in the Model group than in the Control group (*p* < 0.01), and both indices were higher after SSW-H treatment than in the Model group (*p* < 0.01; Figures 3A,B). PCA showed separation of the three groups in the two-dimensional ordination space (PC1 = 21.77%, PC2 = 9.80%; Figure 3C). At the phylum level, the Model group showed a higher relative abundance of Firmicutes and a lower relative abundance of Bacteroidota than the Control group, whereas the overall composition of the SSW-H group more closely resembled that of the Control group (Figure 3D). At the species level (Figure 3E), *Lactobacillus murinus* showed an upward trend in the Model group, whereas *Akkermansia muciniphila*, *Lactobacillus reuteri*, and *Bifidobacterium pseudolongum* were less abundant than in the Control group. Relative to the Model group, the abundance of *Lactobacillus murinus* decreased and those of *Akkermansia muciniphila*, *Lactobacillus reuteri*, and *Bifidobacterium pseudolongum* increased after SSW-H treatment. LEfSe analysis (LDA score >4, *p* < 0.05) identified *Lactobacillus* as a discriminatory genus in the Control group, *Ligilactobacillus* and *Desulfovibrio* in the Model group, and *Akkermansia* in the SSW-H group (Figures 3F,G). Fecal acetic acid, propionic acid, isobutyric acid, butyric acid, isovaleric acid, and valeric acid levels were lower in the Model group than in the Control group (*p* < 0.05), whereas hexanoic acid levels did not differ between the Model and Control groups. SSW-H treatment increased fecal acetic acid, propionic acid, and butyric acid levels relative to those in the Model group (*p* < 0.01; Figure 3H). The serum acetic acid level changed in the same direction (Figure 3I).

### 3.4 FMT distinguished recipient phenotypes associated with different donor sources

**Figure 4:**
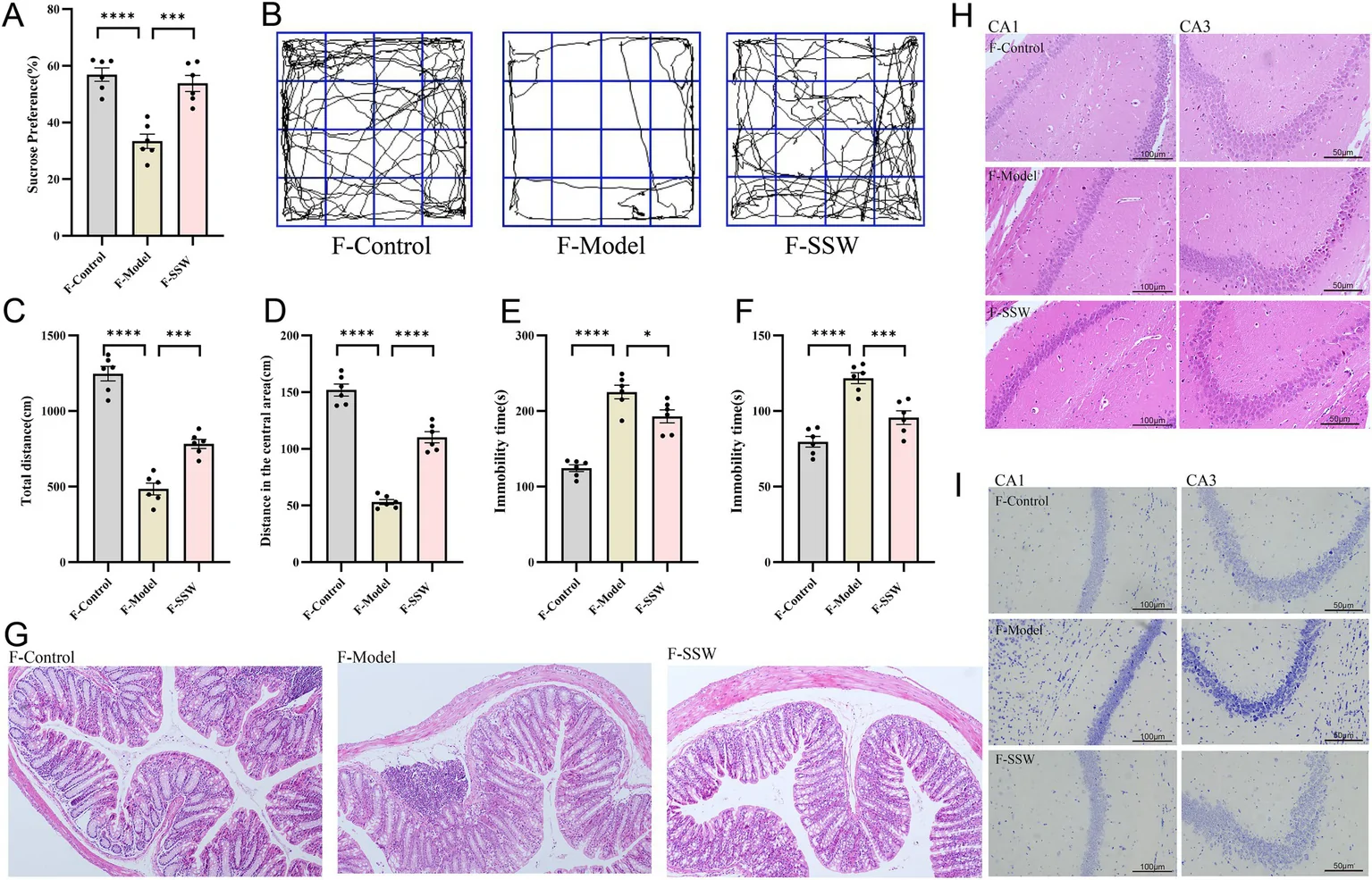
Behavioral and histological analyses of recipient mice after FMT from different donor groups. **(A)** Sucrose preference. **(B)** Representative OFT trajectories. **(C,D)** Total and central-zone distances in the OFT. **(E)** TST immobility time. **(F)** FST immobility time. **(G)** Colonic H&E staining. **(H)** Hippocampal H&E staining. **(I)** Hippocampal Nissl staining. Donor fecal material was obtained from Control, Model, and SSW-H mice. The recipient groups were FMT-Control, FMT-Model, and FMT-SSW (*n* = 6 per group). Behavioral data are presented as mean ± SEM. Statistical procedures and significance symbols are specified in the panels and Section 2.15.

FMT was used to assess whether the microbial states associated with complex donor fecal material were accompanied by distinct recipient phenotypes. Sucrose preference was lower in the FMT-Model group than in the FMT-Control group and higher in the FMT-SSW group than in the FMT-Model group (*p* < 0.05; Figure 4A). Total and central-zone distances in the OFT were lower in the FMT-Model group than in the FMT-Control group and higher in the FMT-SSW group than in the FMT-Model group (*p* < 0.05; Figures 4B–D). TST and FST immobility times were longer in the FMT-Model group than in the FMT-Control group and shorter in the FMT-SSW group than in the FMT-Model group (*p* < 0.05; Figures 4E,F). Colonic and hippocampal histological abnormalities were observed in the FMT-Model group, whereas no evident abnormalities were observed in the FMT-SSW group (Figures 4G–I). Thus, recipients of Model-donor fecal material recapitulated several behavioral and histological features observed after CSD, whereas recipients of SSW-H-donor material did not show comparable abnormalities.

### 3.5 Gut microbial analysis of FMT recipients

**Figure 5:**
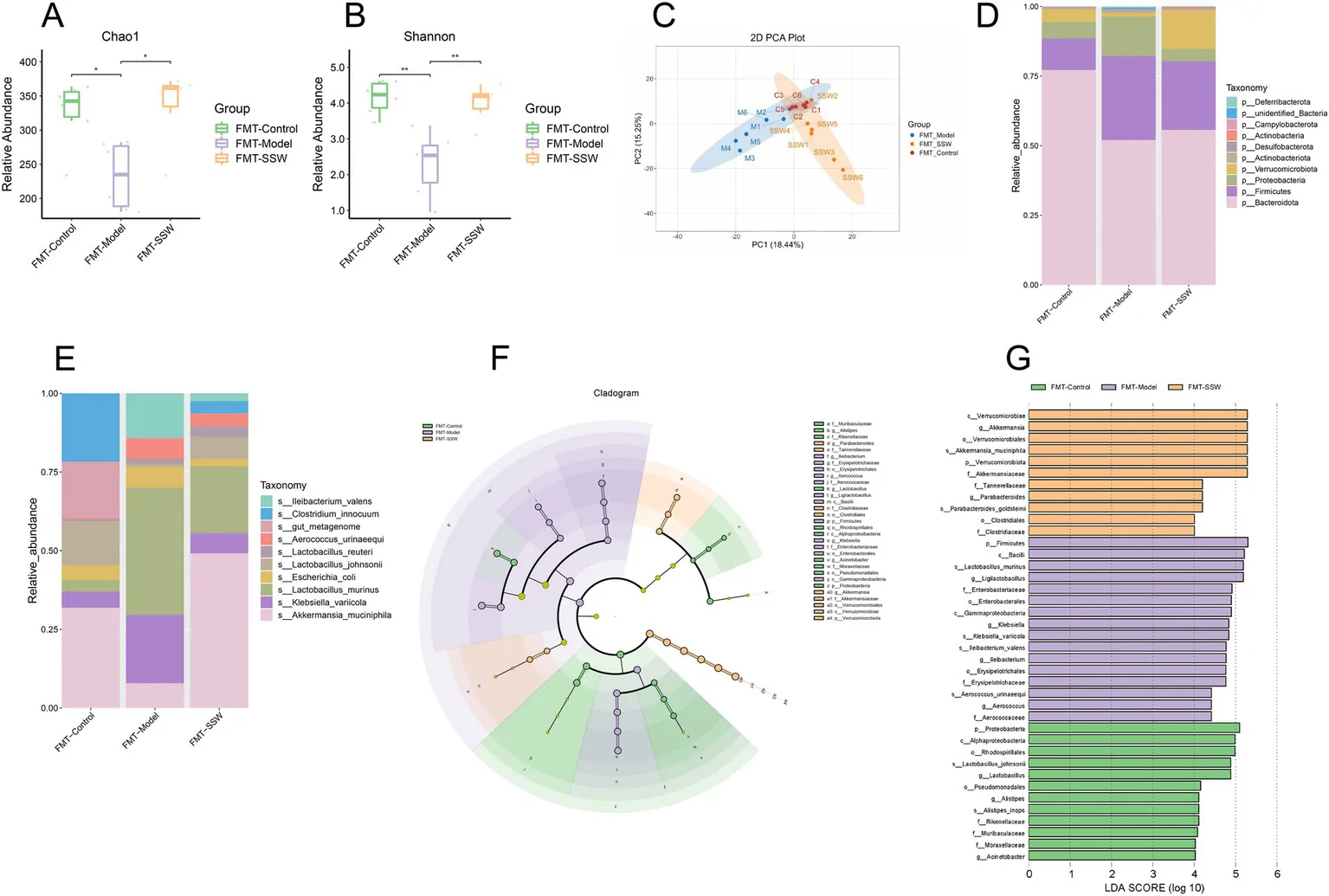
Gut microbial communities in recipient mice after FMT. **(A,B)** Chao1 and Shannon indices. **(C)** PCA ordination. **(D)** Phylum-level community composition. **(E)** Species-level community composition. **(F,G)** LEfSe cladogram and LDA score plot. The mice were categorized into FMT-Control, FMT-Model, and FMT-SSW groups (*n* = 6 per group). Statistical procedures and significance symbols are specified in the panels and Section 2.15.

Fecal samples from the FMT-Control, FMT-Model, and FMT-SSW groups were subjected to 16S rRNA gene sequencing to assess the recipient microbial communities after transplantation. The Chao1 and Shannon indices were lower in the FMT-Model group than in the FMT-Control group and higher in the FMT-SSW group than in the FMT-Model group (Figures 5A,B). PCA showed partial separation among the three groups (Figure 5C). FMT-Model samples were concentrated mainly on the negative side of the ordination axes and were displaced from the FMT-Control group. FMT-SSW samples were generally separated from the FMT-Model cluster, while some were adjacent to the FMT-Control distribution, indicating that recipients of SSW-H-donor material developed a community profile distinct from that of FMT-Model recipients and partly oriented toward the FMT-Control profile. At the phylum level (Figure 5D), the FMT-Model group showed lower abundance of Bacteroidota and higher abundances of Firmicutes and Proteobacteria, whereas the FMT-SSW group showed higher abundances of Bacteroidota and Verrucomicrobiota and a lower abundance of Firmicutes. At the species level, *Akkermansia muciniphila* was less abundant and *Klebsiella variicola* and *Lactobacillus murinus* were more abundant in the FMT-Model group; in the FMT-SSW group, the abundance of these taxa shifted partly toward their abundance levels in the FMT-Control group (Figure 5E). LEfSe identified *Ligilactobacillus*, *Klebsiella*, and Enterobacteriaceae as discriminatory taxa in the FMT-Model group and *Akkermansia*, *Akkermansiaceae*, and *Parabacteroides* as discriminatory taxa in the FMT-SSW group (Figures 5F,G).

### 3.6 Live *Akkermansia muciniphila* supplementation attenuated CSD-associated behavioral and inflammatory abnormalities

**Figure 6:**
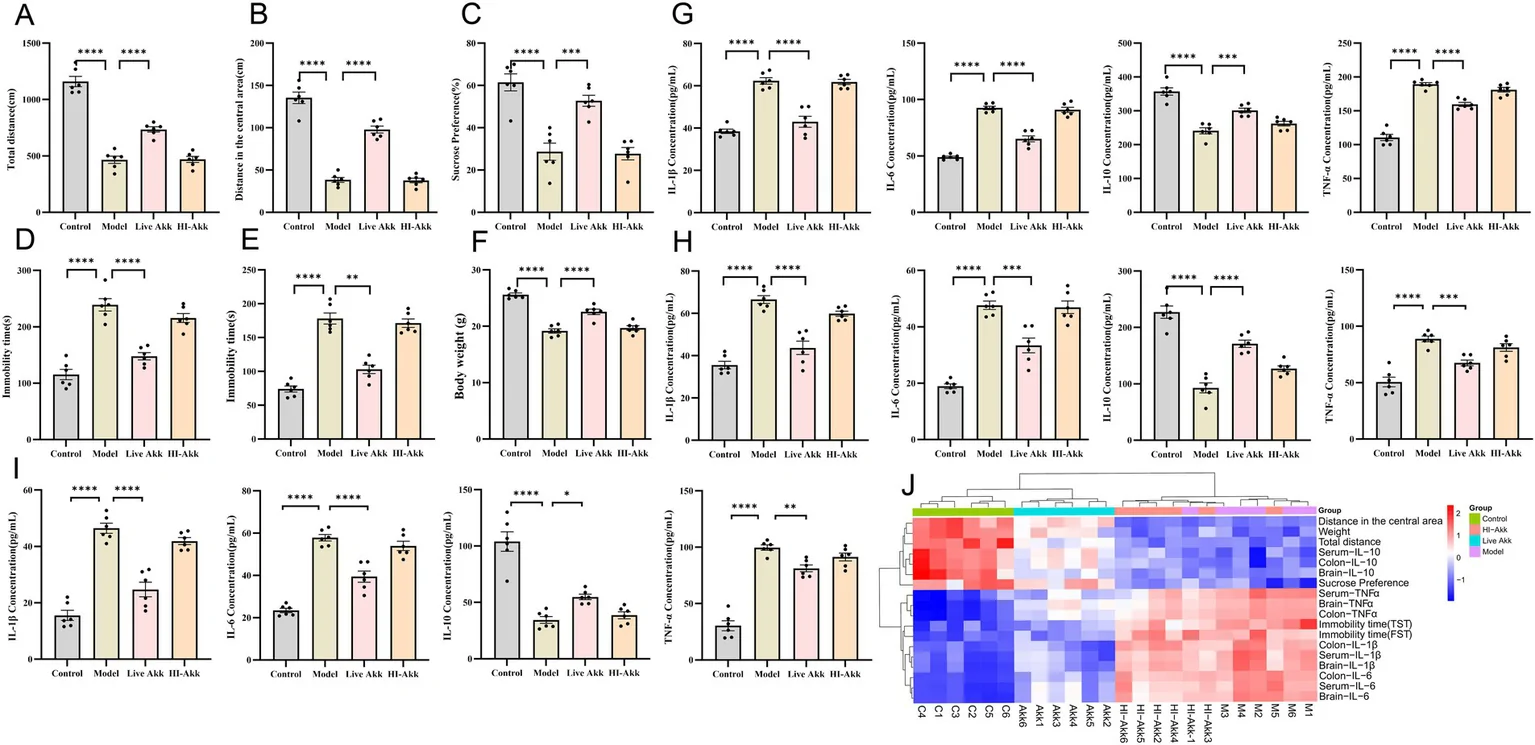
Effects of live and heat-inactivated *Akkermansia muciniphila* on behavioral and inflammatory abnormalities in CSD mice. **(A)** Total distance traveled in the OFT. **(B)** Distance traveled in the central zone of the OFT. **(C)** Sucrose preference. **(D)** TST immobility time. **(E)** FST immobility time. **(F)** Body weight. **(G–I)** IL-1β, IL-6, IL-10, and TNF-α concentrations in the colon homogenate, serum, and hippocampal homogenate, respectively. **(J)** Hierarchical clustered heatmap of Z-score-normalized behavioral, body-weight, and cytokine measurements. Rows represent individual variables, and columns represent individual mice. Red and blue indicate values above and below the overall mean for each variable, respectively. Mice were categorized into Control, Model, CSD + Live Akk, and CSD + HI-Akk groups (*n* = 6 per group). Live Akk denotes live *A. muciniphila*, and HI-Akk denotes heat-inactivated *A. muciniphila*. Data in A–I are presented as mean ± SEM; each point represents one biologically independent mouse. Statistical procedures are described in Section 2.15. *p* < 0.05, *p* < 0.01, *p* < 0.001, and *p* < 0.0001 for the comparisons indicated in the panels.

To further assess the potential contribution of *A. muciniphila* to the phenotype associated with SSW treatment, we compared the effects of live and heat-inactivated *A. muciniphila* in CSD mice. Relative to the Control group, Model mice showed shorter total and central-zone distances in the OFT, lower sucrose preference and body weight, and longer immobility times in the TST and FST (*p* < 0.05, Figures 6A–F). Live *A. muciniphila* supplementation increased total and central-zone distances, sucrose preference, and body weight and reduced TST and FST immobility relative to the Model group (*p* < 0.05). In contrast, the corresponding behavioral measures in the HI-Akk group generally remained closer to those in the Model group. CSD was also accompanied by higher IL-1β, IL-6, and TNF-α concentrations and lower IL-10 concentrations in the colon, serum, and hippocampus than in the Control group (*p* < 0.05, Figures 6G–I). Live *A. muciniphila* reduced IL-1β, IL-6, and TNF-α concentrations and increased IL-10 concentrations in all three sample types relative to the Model group (*p* < 0.05), whereas the heat-inactivated preparation did not show a comparable overall pattern. Hierarchical clustering of the Z-score-normalized behavioral, body-weight, and cytokine data further distinguished the Control and Model profiles; samples from the Live Akk group showed an overall pattern shifted toward the Control profile, whereas the HI-Akk group remained more similar to the Model group (Figure 6J). These findings provide additional intervention-based support for a functional contribution of *A. muciniphila* to the behavioral and inflammatory changes associated with the SSW-related intestinal state.

### 3.7 Serum metabolomics identified host metabolic responses associated with SSW treatment

**Figure 7:**
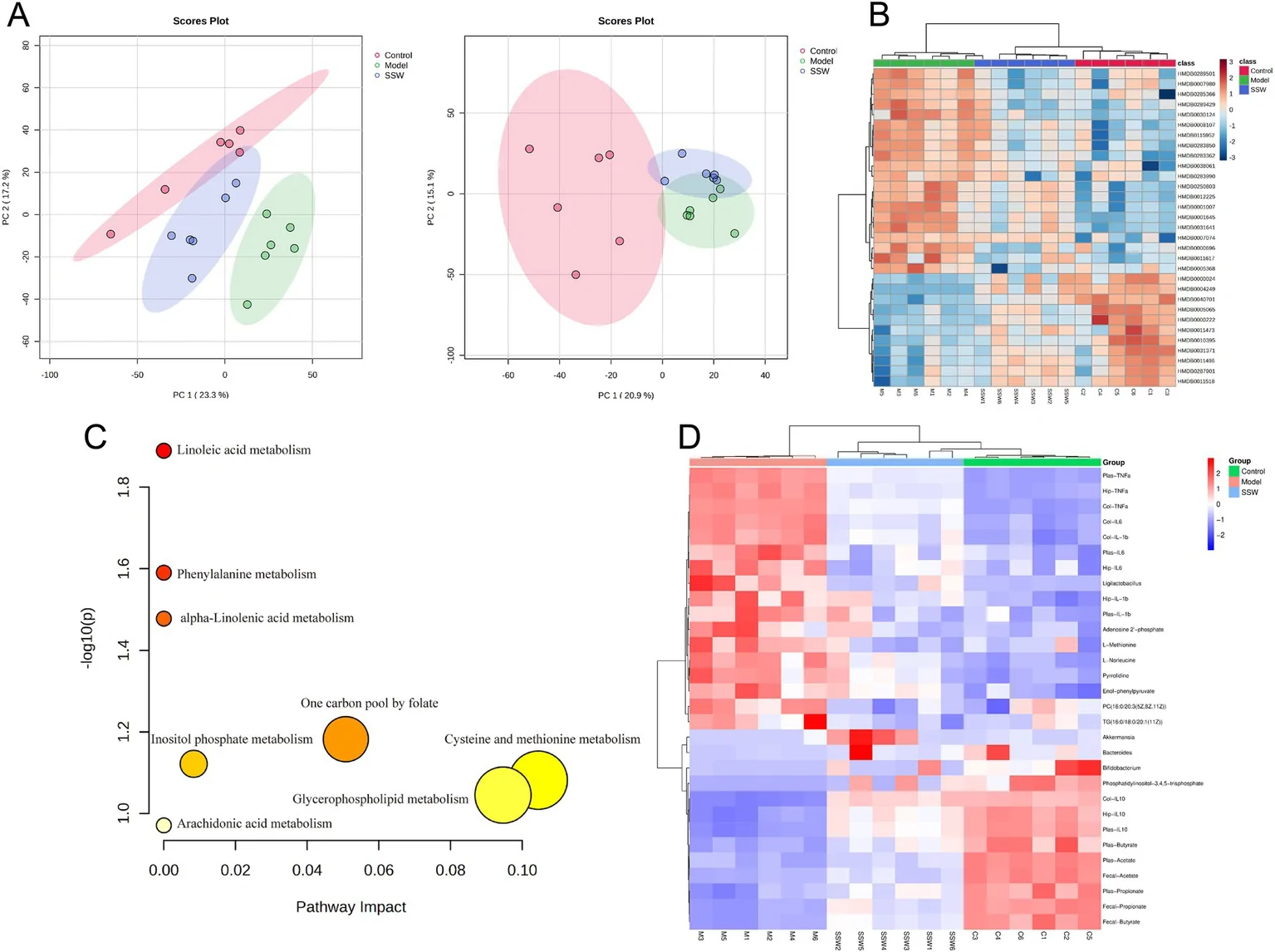
Serum untargeted metabolomics and integrated patterns associated with SSW treatment and inflammation **(A)** PCA ordination. **(B)** Clustered heatmap of differential metabolic features. **(C)** Pathway enrichment of differential metabolic features. **(D)** Integrated clustered heatmap of differential taxa, SCFAs, inflammatory markers, and differential metabolic features. Differential metabolic features were selected using VIP > 1 and *p* < 0.05. The mice were categorized into Control, Model, and SSW-H groups. Serum untargeted metabolomics was performed with *n* = 6 mice per group.

Serum untargeted metabolomics revealed a tendency toward separation of the Control, Model, and SSW-H groups in the PCA space, consistent with the altered circulating metabolic profile after CSD and partial reorientation after SSW-H treatment (Figure 7A). Subsequently, pairwise OPLS-DA models were constructed. In positive- and negative-ion modes, Control and Model serum samples were clearly separated, with R2Y values of 0.971 and 0.994 and Q2 values of 0.884 and 0.784, respectively. Similar separation was observed between the SSW-H and Model groups, with R2Y values of 0.992 and 0.991 and Q2 values of 0.869 and 0.661, respectively (Supplementary Figure S2). Using VIP > 1 and *p* < 0.05, 31 differential metabolites were common to the Control-versus-Model and SSW-H-versus-Model comparisons (Figure 7B; Supplementary Table S6). Nineteen serum metabolites showed a significant shift after SSW-H treatment (*p* < 0.05). The levels of triricinolein and phosphatidylinositol 3,4,5-trisphosphate increased, whereas those of the other 17 metabolites, including TG(16:0/18:0/20:1(11Z)) and prenol, decreased. Enrichment analysis of the SSW-H-responsive metabolites identified linoleic acid metabolism, phenylalanine metabolism, alpha-linolenic acid metabolism, inositol phosphate metabolism, arachidonic acid metabolism, folate-mediated one-carbon metabolism, glycerophospholipid metabolism, and cysteine and methionine metabolism (Figure 7C). This pattern was consistent with the broad modulation of lipid, amino-acid, and one-carbon metabolic features rather than selective activation of a single pathway. The combined heatmap showed broadly concordant groupwise patterns among microbial taxa, SCFAs, inflammatory cytokines, and differential metabolic features (Figure 7D). This visualization was descriptive and was not intended to represent formal cross-platform multi-omics integration. SSW-H was associated with higher relative abundance of *Akkermansia*, lower abundance of *Ligilactobacillus*, partial restoration of fecal SCFA and serum acetic acid levels, lower inflammatory cytokine concentrations, and partial normalization of several lipid- and amino-acid–related candidate metabolite levels.

### 3.8 Hippocampal transcriptomics revealed partial normalization of CSD-associated immune and neural-function signatures

**Figure 8:**
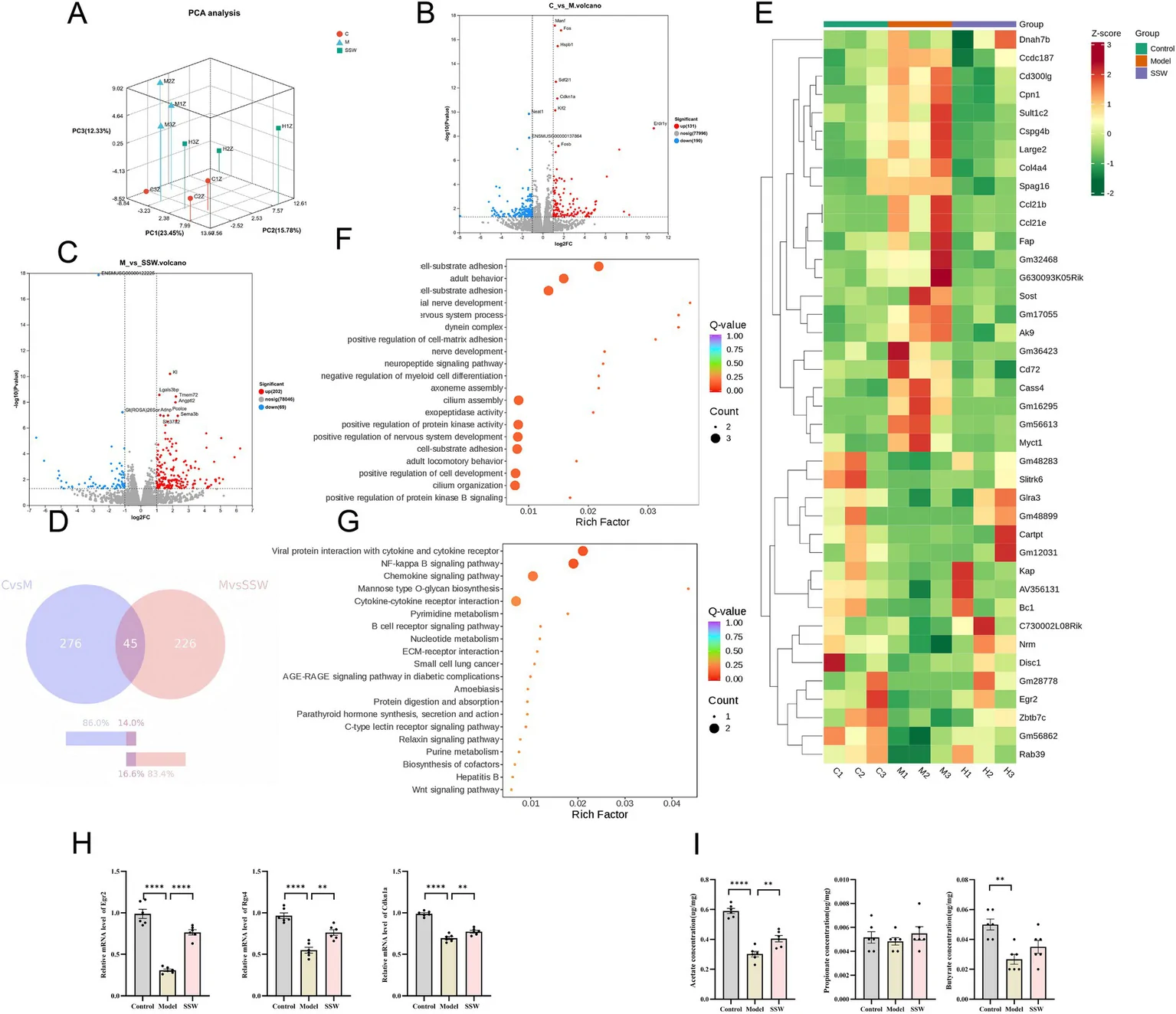
Hippocampal transcriptomics, RT-qPCR validation of candidate genes, and hippocampal SCFAs. **(A)** PCA of hippocampal transcriptomes. **(B,C)** Volcano plots for the Control versus Model and Model versus SSW-H groups, respectively. **(D)** Venn diagram of overlapping candidate DEGs. **(E)** Heatmap of overlapping DEG expression. **(F,G)** GO and KEGG enrichment analyses. **(H)** RT-qPCR results for *Egr2*, *Rgs4*, and *Cdkn1a*. **(I)** Hippocampal acetic acid, propionic acid, and butyric acid levels. RT-qPCR and SCFA data are presented as mean ± SEM; each point represents one biologically independent mouse. Relative expression was calculated using the 2^-*ΔΔCt*^ method, with the Control group normalized to 1. Statistical procedures and significance symbols are specified in the panels and Section 2.15. Hippocampal transcriptomics used *n* = 3 per group; RT-qPCR and hippocampal SCFA analyses used *n* = 6 per group. DEGs were defined using |log2 fold change| ≥ 1 and adjusted *p* value < 0.05.

Hippocampal transcriptomes were analyzed in the Control, Model, and SSW-H groups. PCA showed partial separation among the three groups (Figure 8A). Using DESeq2 and the stated criteria of |log2 fold change| ≥ 1 and adjusted *p* value < 0.05, 321 DEGs, including 190 upregulated and 131 downregulated genes, were identified in the Model-versus-Control comparison. The SSW-H-versus-Model comparison yielded 271 DEGs, including 69 upregulated and 202 downregulated genes (Figures 8B,C). Forty-five DEGs overlapped between the two comparisons (Figure 8D). After removing unannotated genes, the final candidate set was displayed in an expression heatmap (Figure 8E). GO enrichment was concentrated in chemokine activity, lymphocyte and monocyte chemotaxis, cell migration, and regulation of myeloid-cell differentiation (Figure 8F). KEGG enrichment included the NF-κB signaling pathway, chemokine signaling pathway, cytokine–cytokine receptor interaction, and C-type lectin receptor signaling pathway (Figure 8G). *Egr2*, *Rgs4*, and *Cdkn1a* were selected for RT-qPCR validation on the basis of transcriptomic direction, statistical support, and functional relevance. All three genes showed lower expression in the Model group and higher expression after SSW-H treatment in the RNA-sequencing data, and RT-qPCR confirmed the overall direction of change (Figure 8H). Hippocampal acetic acid and butyric acid levels were lower in the Model group, whereas acetic acid levels were higher in the SSW-H group. Propionic acid and butyric acid levels did not show stable differences between the SSW-H and Model groups (Figure 8I). These findings were associated with partial restoration of hippocampal transcriptional signatures related to immune regulation and stress adaptation, together with normalization of hippocampal acetic acid.

## 4 Discussion

CSD reduced sucrose preference and open-field activity, prolonged immobility in the TST and FST, reduced body weight, disrupted colonic mucosal architecture, altered hippocampal neuronal morphology, and increased pro-inflammatory cytokine levels in the colon, serum, and hippocampus. SSW improved most of these measures and findings, with the most consistent effects observed at 5.0 g/kg. The breadth of the response indicates that instead of reflecting a single pathway-specific action, the effects of SSW encompassed several domains affected by CSD. Zhang et al. likewise observed concurrent anxiety-like behavior, gut microbial changes, and serum metabolic abnormalities in sleep-deprived animals (Zhang N. et al., 2024). In the rs-fMRI analysis, CSD-associated differences in the VBM, ALFF, and ReHo in the hippocampus, amygdala, and other regions were attenuated after SSW-H treatment. These imaging findings should remain exploratory because only six mice per group were scanned, and all data were acquired under isoflurane anesthesia. Larger imaging cohorts will be needed to establish the robustness and biological interpretability of these findings.

Our microbiome analysis focused on whether changes in community structure were accompanied by changes in microbial metabolite levels. CSD reduced alpha diversity, as reflected by the Chao1 and Shannon indices, and shifted the overall community composition away from the Control profile; the SSW-H distribution shifted toward the Control profile. Karl et al. also observed reduced microbial richness with unchanged intestinal permeability after short-term sleep restriction in humans (Karl et al., 2023), indicating that exposure duration, model intensity, and host condition may modify the microbial response. *Akkermansia* was enriched in the SSW-H group, whereas *Ligilactobacillus* and *Desulfovibrio* were more prominent in the Model group. Associations between *Akkermansia*, mucus-layer homeostasis, and metabolic health have been reported in both animal and human studies (Depommier et al., 2019; Everard et al., 2013). SCFA measurements provided a complementary metabolic readout. Fecal acetic acid, propionic acid, and butyric acid levels were all reduced by CSD and increased after SSW-H treatment, whereas acetic acid levels showed the most consistent direction of change in serum and hippocampal tissue. Fecal concentrations reflect net luminal availability; serum concentrations represent circulating exposure; and hippocampal concentrations reflect local tissue distribution; these measures were not physiologically interchangeable. Importantly, the present measurements do not demonstrate that intestinally derived SCFAs crossed the blood–brain barrier or that the acetate detected in hippocampal tissue originated from the gut. Stable-isotope tracing will be required to resolve the source, systemic transport, and central fate of SCFAs in future studies. Thus, the data support correction of fecal SCFA depletion by SSW-H and a concordant direction of changes in the acetic acid levels across the intestinal, circulatory, and cerebral compartments. However, the direct contribution of acetic acid to reduced hippocampal inflammation or behavioral recovery is difficult to ascertain without supplementation or receptor-blockade experiments.

The FMT experiment provides intervention-based support for the involvement of a donor-associated intestinal state, but it does not identify the specific component of the transplanted fecal material responsible for the recipient phenotypes. In the present study, recipient mice were not subjected to CSD and did not receive SSW directly, yet transplantation of Model-donor fecal material was followed by reduced sucrose preference, lower activity, longer immobility, and colonic and hippocampal histological abnormalities. Recipients of SSW-H-donor material more closely resembled the FMT-Control group. The recipient communities did not reproduce every donor feature, but instead showed group-specific ecological patterns after transplantation. This is compatible with the re-establishment of a recipient ecosystem rather than simple one-to-one transfer of individual taxa. Kelly et al. observed neurobehavioral changes after transferring fecal microbiota from patients with depression to rats, and Li et al. reported partial transfer of anxiety- and depression-like phenotypes from chronic-stress donors to recipient mice (Kelly et al., 2016; Li et al., 2019). Other studies have linked dysbiosis to complement C3-associated abnormal synaptic pruning (Hao et al., 2024), intestinal permeability and 5-HT expression in an alcohol-related model (Li D. et al., 2024), and endocannabinoid signaling, host metabolism, and hippocampal neurogenesis (Chevalier et al., 2020; Zheng et al., 2016; He et al., 2024). In the present study, all donors were obtained from the same CSD experiment, allowing direct comparison of the Model- and SSW-H-associated donor states. Taken together, these findings indicate that fecal material from the different donor groups was associated with distinct recipient phenotypes. However, because complete fecal suspensions were transferred, the relative contributions of bacteria, metabolites, and other transferred components cannot be distinguished. The FMT findings therefore should not be interpreted as evidence that the effects of SSW are specifically dependent on bacterial transfer.

The targeted *A. muciniphila* experiment provided additional support for the functional relevance of the microbial changes observed after SSW treatment. Live *A. muciniphila* improved sucrose preference and open-field activity, shortened TST and FST immobility, and attenuated the pro-inflammatory cytokine profile in the colon, serum, and hippocampus. In contrast, heat-inactivated *A. muciniphila* did not show a comparable overall pattern, and clustered measurements from the Live Akk group shifted toward the Control profile. These findings support *A. muciniphila* as a candidate contributor to the intestinal state associated with SSW treatment rather than establishing it as a sole or necessary mediator. Moreover, because SCFA supplementation and receptor-blockade experiments were not performed, the present data do not establish whether SCFAs are required downstream effectors of *A. muciniphila*.

The changes associated with SSW were not limited to the intestine. Serum metabolic features that shifted after treatment included enrichment in linoleic acid, alpha-linolenic acid, arachidonic acid, glycerophospholipid, phenylalanine, one-carbon, cysteine, and methionine metabolism. Integrated metabolomic and proteomic analyses by Hu et al. showed broad disruption of fatty-acid, amino-acid, and energy metabolism after sleep deprivation (Hu et al., 2021), and a large untargeted metabolomics study in humans revealed distinct circulating profiles associated with sleep duration, efficiency, and sleep-disordered breathing (Zhang Y. et al., 2025). Against this background, the pathway enrichments observed in the present study can be more appropriately interpreted as broad reorientation of CSD-associated systemic metabolic disturbances after SSW-H treatment than as specific activation of a single pathway. Hippocampal transcriptional changes were enriched in annotations related to immune processes, including chemokine activity, myeloid-cell differentiation, cytokine–cytokine receptor interaction, and NF-κB signaling. Gaine et al. reported that sleep deprivation had persistent effects on hippocampal transcription (Gaine et al., 2021), and spatial transcriptomic data from Vanrobaeys et al. showed that transcriptional responses differ among brain regions and hippocampal subregions (Vanrobaeys et al., 2023). The expression of Egr2, Rgs4, and Cdkn1a changed in the same direction in RNA-sequencing and RT-qPCR analyses. Therefore, these three genes can be best considered representative markers of an adjusted hippocampal transcriptional state after SSW-H treatment rather than components of a newly established regulatory pathway. These enrichment results reflect transcript-level associations and should not be interpreted as direct evidence of activation or inhibition of the corresponding pathways. Because validation was limited to mRNA expression and no protein-level or pathway-intervention experiments were performed, the present data do not establish a causal relationship between SCFA changes, Egr2/Rgs4/Cdkn1a expression, and NF-κB or chemokine signaling. Xu et al. also observed concurrent intestinal and hippocampal metabolic abnormalities in a chronic-stress model (Xu et al., 2022), indicating that peripheral and central changes can occur in parallel without resolving their causal order. The same limitation is applicable here: temporal sampling and targeted interventions will be required to define the relationships among the microbiota, SCFAs, serum metabolome, and hippocampal transcriptome.

Previous SSW studies have focused mainly on colitis. The present study extended the experimental context to CSD and documented concurrent changes in mood-related behavior, hippocampal histology, circulating metabolites, and the donor-associated intestinal state assessed by FMT. Several studies of traditional medicinal formulas have similarly integrated gastrointestinal, behavioral, and microbial outcomes. Zuojin Pill was shown to improve depression-like behavior and gastrointestinal dysfunction in chronically stressed mice (Wang Y. et al., 2023), whereas Xiao-Chai-Hu-Tang showed microbiota-dependent modulation of TLR4/myeloid differentiation primary response 88 (MyD88)/NF-κB signaling in a tumor model with depressive symptoms (Shao et al., 2021). A recent review described a shift in natural-product antidepressant research from isolated central targets toward integrated microbiota–metabolism–immunity–neuroplasticity models (Lu et al., 2024). The present study addresses a more specific stressor—sleep deprivation—rather than chronic unpredictable stress or lipopolysaccharide-induced inflammation. From a traditional Chinese medicine perspective, the established use of SSW for chronic diarrhea associated with spleen–kidney yang deficiency provided an additional rationale for its selection, particularly in view of the intestinal abnormalities observed under CSD. Nevertheless, because no traditional Chinese medicine syndrome model or syndrome-based assessment was incorporated into the present study, these findings should not be interpreted as evidence of a direct correspondence between CSD and spleen–kidney yang deficiency.

However, several limitations of the study require consideration. Sleep architecture was not directly recorded by electroencephalography/electromyography; therefore, the extent of sleep loss and rebound sleep was not objectively quantified. The FMT experiment used complete fecal suspensions and therefore could not distinguish the effects of bacteria from those of metabolites or other transferred components. Causality was not tested by monocolonization with a candidate strain such as Akkermansia or by independent supplementation with an SCFA such as acetate. The temporal sequence and causal direction linking hippocampal transcriptomic changes, serum metabolic features, and SCFA alterations also remain unresolved. In addition, although findings from the microbiome, SCFA, serum metabolomic, hippocampal transcriptomic, and rs-fMRI analyses were considered together, formal cross-platform multi-omics modeling was not performed. The current cross-layer comparison should therefore be regarded as descriptive, and future studies with harmonized sampling and prespecified integrative analyses will be required to establish robust relationships across these data layers.

## 5 Conclusion

SSW ameliorated CSD-associated behavioral abnormalities, colonic and hippocampal injury, and inflammatory responses in mice, with the high dose producing the most consistent effects. These improvements were accompanied by shifts in gut microbial composition and partial restoration of fecal, serum, and hippocampal SCFA profiles. Recipients of FMT developed distinct behavioral, histological, and microbial phenotypes according to whether donor material originated from Control, Model, or SSW-H mice. These findings support the involvement of a donor-associated intestinal state in recipient responses, but do not establish that bacterial transfer alone accounted for the observed effects. Serum metabolomics and hippocampal transcriptomics provided complementary evidence of treatment-associated systemic and central molecular changes, whereas the rs-fMRI findings were considered exploratory because of the limited imaging sample size. The present study did not establish a complete causal pathway, and the respective contributions of individual microbial taxa, metabolites, and downstream host processes require targeted validation.

## Data Availability

The datasets presented in this study can be found in online repositories. The names of the repository/repositories and accession number(s) can be found in the article/Supplementary material.
