# Supplementary material for: Sishen wan modulates gut microbial and short-chain fatty-acid imbalances and ameliorates behavioral and inflammatory abnormalities in mice with chronic sleep deprivation
Source: Front Microbiol. 2026 Sep 3;17:1944977. doi: 10.3389/fmicb.2026.1944977 (PMC13582583; doi:10.3389/fmicb.2026.1944977)
Supplement: Supplementary file 1 [file Supplementary_file_1.docx]

**Sishen Wan modulates gut microbial and short-chain fatty-acid imbalances and ameliorates behavioral and inflammatory abnormalities in mice with chronic sleep deprivation**

Yu Jiao^1,2†^, Yichun Zhao^3†^, Zhongqiang Zhang^1^, Hui Sun^1^, Yaoai Wang^1^, Xueke Bai^1^, Chengyu Piao^1^, Ning Zhang^1*^, Xia Lei^3*^, Hongdan Xu^4*^

^1^College of Pharmacy, Heilongjiang University of Chinese Medicine, Harbin, Heilongjiang, China.

^2^Heilongjiang Provincial key Laboratory of Precise Diagnosis and Neuropsychological Regulation of Mental Disorders,Qiqihar, Hleilongjiang, China.

^3^Wuxi Affiliated Hospital of Nanjing University of Chinese Medicine, Nanjing University of Chinese Medicine, Wuxi, Jiangsu, China; Jiangsu CM Clinical Innovation Center of Degenerative Bone & Joint Disease, Wuxi, Jiangsu, China.

^4^Wuxi Affiliated Hospital of Nanjing University of Chinese Medicine, Nanjing University of Chinese Medicine, Wuxi, Jiangsu, China; Department of Pharmacy, Wuxi Health higher Vocational and Technical School, Wuxi, Jiangsu, China.

**^†^**These authors contributed equally to this work and share first authorship: Yu Jiao^1^**^†^**, Yichun Zhao^2^**^†^**

**^*^Correspondence:** Ning Zhang [zhangning0454@163.com](mailto:zhangning0454@163.com); Xia Lei [wxzy094@njucm.edu.cn](mailto:wxzy094@njucm.edu.cn); Hongdan Xu [xuhd8431@126.com](mailto:xuhd8431@126.com).

**Supplementary Methods S1. UPLC–QTOF–MS-Based Chemical Characterization of Sishen Wan**

An appropriate amount of Sishen Wan (SSW) was pulverized and passed through a 100-mesh sieve. The powdered sample (1.0 g) was accurately weighed and transferred to a stoppered conical flask, followed by the addition of 50 mL of 70% acetonitrile. The mixture was sonicated at 360 W and 40 kHz for 30 min, mixed thoroughly, and filtered. The subsequent filtrate was centrifuged at 12,000 rpm for 10 min and passed through a 0.22-μm microporous membrane filter before analysis.

Untargeted chemical profiling of SSW was performed using ultra-performance liquid chromatography coupled with quadrupole time-of-flight mass spectrometry (UPLC–QTOF–MS). Chromatographic separation was conducted on an ACQUITY HSS T3 column (2.1 mm × 100 mm, 1.8 μm). The mobile phases consisted of 0.1% formic acid in water (solvent A) and acetonitrile (solvent B). The column temperature was maintained at 40 °C, the flow rate was set at 0.3 mL/min, and the injection volume was 5 μL.

The gradient elution program was as follows: 0–2 min, 5% B; 2–7 min, 30% B; 7–10 min, 30–60% B; 10–13 min, 60–80% B; 13–17 min, 90% B; 17–20 min, 100% B; and 20–23 min, 5% B.

Mass spectrometric detection was performed using an X500B QTOF mass spectrometer equipped with an electrospray ionization source operated in both positive- and negative-ion modes. Data were acquired using information-dependent acquisition (IDA). The full-scan MS range was set to m/z 100–1200, and the MS/MS scan range was set to m/z 50–1200. High-purity nitrogen was used as the collision and nebulizing gas. The ion spray voltage was set to +5500 V in positive-ion mode and −5500 V in negative-ion mode. The collision energy was set to +10 V and −10 V in positive- and negative-ion modes, respectively. The declustering potential was set to +100 V and −100 V, respectively. Curtain gas pressure was set to 35 psi, while nebulizer gas and auxiliary gas pressures were both set to 60 psi.

Candidate compounds were annotated on the basis of retention time, accurate molecular mass, adduct ion forms, and MS/MS fragmentation information. Candidate annotations obtained from positive- and negative-ion modes were integrated, and duplicate annotations were removed before subsequent chemical characterization. The complete list of 92 putatively annotated constituents is provided in Table S1.

**Table S1. Putatively annotated constituents of Sishen Wan identified by UPLC–QTOF–MS in positive- and negative-ion modes**

| **No.** | **Name** | **Rt (min)** | **Precursor m/z** | **Adduct** | **Formula** | **Error/ppm** | **MS/MS fragments** |
| --- | --- | --- | --- | --- | --- | --- | --- |
| 1 | Glycerophosphocholine | 0.922 | 258.11023 | [M+H]+ | C8H20NO6P | 0.5 | 104.10732; 125.00036; 86.09674; 60.08118 |
| 2 | Sucrose | 0.95 | 365.10616 | [M+Na]+ | C12H22O11 | 2 | 365.10621; 203.05333; 185.04251; 245.05185 |
| 3 | Trigonelline | 0.962 | 138.05479 | [M+H]+ | C7H7NO2 | -1.19 | 92.04949; 65.03869; 78.03397; 94.06516 |
| 4 | Xanthine | 1.173 | 153.04064 | [M+H]+ | C5H4N4O2 | -0.4 | 55.02948; 110.03555; 81.00874 |
| 5 | Salsolinol | 1.248 | 180.10182 | [M+H]+ | C10H13NO2 | -0.47 | 115.05452; 117.06994; 91.05468; 145.06515 |
| 6 | Kynurenic acid | 5.258 | 190.04988 | [M+H]+ | C10H7NO3 | 0.05 | 128.05088; 89.03922; 116.05056; 144.04299 |
| 7 | Chlorogenic acid | 5.637 | 355.10233 | [M+H]+ | C16H18O9 | -0.08 | 163.03931; 145.02917; 135.04469; 117.03419 |
| 8 | Riboflavin | 6.215 | 377.14687 | [M+H]+ | C17H20N4O6 | 3.47 | 243.08869; 172.08812; 198.06692; 69.03366 |
| 9 | Corymboside | 6.452 | 565.15499 | [M+H]+ | C26H28O14 | -0.34 | 379.08189; 409.09250; 427.10287; 325.07157 |
| 10 | Schaftoside | 6.452 | 587.13884 | [M+Na]+ | C26H28O14 | 2.92 | 569.12890; 551.12005; 479.09719; 509.10688 |
| 11 | Reticuline | 6.494 | 330.17054 | [M+H]+ | C19H23NO4 | 1.68 | 192.10284; 137.06001; 177.07920; 175.07631 |
| 12 | 6-Methylcoumarin | 6.547 | 161.06013 | [M+H]+ | C10H8O2 | 2.63 | 105.07070; 115.05449; 79.05489; 63.02354 |
| 13 | Psoralen | 6.57 | 187.03865 | [M+H]+ | C11H6O3 | -1.71 | 131.04906; 77.03866; 115.05440; 103.05468 |
| 14 | (E)-3-[4-(beta-D-glucopyranosyloxy)-5-benzofuranyl]acrylic acid | 6.57 | 389.0848 | [M+Na]+ | C17H18O9 | 1.28 | 389.08393; 227.03231; 185.04280; 345.09608 |
| 15 | Quercetin | 6.663 | 303.05032 | [M+H]+ | C15H10O7 | 1.29 | 153.01962; 229.05025; 137.02455; 165.01935 |
| 16 | Eriodictyol | 6.863 | 289.07095 | [M+H]+ | C15H12O6 | 0.99 | 153.01852; 163.03975; 135.04581; 89.03927 |
| 17 | 3'-Hydroxypuerarin | 7.005 | 433.11333 | [M+H]+ | C21H20O10 | 0.94 | 283.06158; 313.07201; 337.07212; 323.09305 |
| 18 | Isorhamnetin | 7.07 | 317.06594 | [M+H]+ | C16H12O7 | 1.14 | 317.06607; 302.04280; 153.01847; 274.04748 |
| 19 | Isoliquiritigenin | 7.116 | 257.08093 | [M+H]+ | C15H12O4 | 0.37 | 137.02399; 91.05494; 119.04941 |
| 20 | Genistein | 8 | 271.06038 | [M+H]+ | C15H10O5 | 1.03 | 153.01914; 215.07131; 243.06663; 91.05503 |
| 21 | 2-Methylquinazolin-4-ol | 8.29 | 161.0706 | [M+H]+ | C9H8N2O | -2.11 | 77.03965; 92.05012; 120.04487; 116.05030 |
| 22 | Daidzein | 9.111 | 255.0652 | [M+H]+ | C15H10O4 | 0.06 | 255.06479; 199.07591; 137.02399; 152.06261 |
| 23 | Kaempferol | 10.184 | 287.05545 | [M+H]+ | C15H10O6 | 1.52 | 153.01934; 121.02939; 165.01808; 258.05320 |
| 24 | Farrerol | 10.758 | 301.10668 | [M+H]+ | C17H16O5 | -1.23 | 181.05051; 119.04845; 147.04508; 301.10803 |
| 25 | Limonin | 11.153 | 471.20097 | [M+H]+ | C26H30O8 | -0.79 | 161.06004; 425.19573; 471.20078; 95.01310 |
| 26 | Licocoumarone | 11.264 | 341.13885 | [M+H]+ | C20H20O5 | 1.47 | 149.02406; 163.03978; 341.13906; 205.08685 |
| 27 | Fragransin B2 | 11.321 | 387.17958 | [M-H2O+H]+ | C22H28O7 | -1.64 | 181.08721; 167.07163; 201.09228; 158.07423 |
| 28 | Malabaricanol | 11.69 | 327.15965 | [M-H2O+H]+ | C20H24O5 | 1.72 | 137.06027; 151.07599; 203.10729; 91.05480 |
| 29 | Bavachin | 11.832 | 325.14279 | [M+H]+ | C20H20O4 | -1.99 | 149.02315; 147.04405; 121.02874; 167.03424 |
| 30 | Isoevodiamine | 11.977 | 304.14431 | [M+H]+ | C19H17N3O | -0.42 | 134.06027; 161.07133; 144.08121; 171.09211 |
| 31 | Gomisin D | 12.248 | 553.20562 | [M+Na]+ | C28H34O10 | 2.18 | 507.19938; 423.14250; 391.11676; 535.19536 |
| 32 | Gomisin J | 12.363 | 389.19606 | [M+H]+ | C22H28O6 | 0.5 | 227.07089; 287.09197; 288.10012; 255.06615 |
| 33 | Corylin | 12.605 | 321.11123 | [M+H]+ | C20H16O4 | -2.82 | 137.02340; 279.06550; 305.08117; 321.11382 |
| 34 | 2-Nonyl-4(1H)-quinolone | 12.626 | 272.20116 | [M+H]+ | C18H25NO | 0.99 | 272.20076; 159.06856; 172.07635; 130.06571 |
| 35 | Angeloylgomisin H | 13.164 | 483.23744 | [M-H2O+H]+ | C28H36O8 | -0.6 | 83.04956; 451.21279; 395.15106; 414.16905 |
| 36 | Myrislignan | 13.249 | 357.16983 | [M-H2O+H]+ | C21H26O6 | 0.5 | 193.08644; 133.06520; 165.09118; 118.04179 |
| 37 | LPC 18:2 | 13.651 | 520.33963 | [M+H]+ | C26H50NO7P | -0.26 | 184.07325; 104.10726; 520.33837; 86.09677 |
| 38 | Raphidecursinol B | 13.671 | 441.18869 | [M+Na]+ | C23H30O7 | 0.72 | 441.18739; 216.07653; 201.05291; 213.08953 |
| 39 | Grandisin | 13.979 | 433.222 | [M+H]+ | C24H32O7 | -0.18 | 181.08725; 247.13325; 216.11621; 195.10344 |
| 40 | 9-Oxotournefortiolide | 13.984 | 247.13317 | [M+H]+ | C15H18O3 | 1.21 | 216.11506; 173.09682; 201.09184; 129.07061 |
| 41 | alpha-Linolenic acid | 14.267 | 279.23213 | [M+H]+ | C18H30O2 | 0.98 | 67.05489; 81.07051; 95.08612; 79.05513 |
| 42 | Schisanhenol | 14.452 | 403.21137 | [M+H]+ | C23H30O6 | -0.36 | 302.11506; 340.16705; 287.09201; 301.10755 |
| 43 | 1-Methyl-2-undecyl-4(1H)-quinolone | 15.395 | 314.2462 | [M+H]+ | C21H31NO | -5.22 | 173.08331; 314.25114; 186.09106; 158.06017 |
| 44 | Benzaldehyde | 15.893 | 107.04917 | [M+H]+ | C7H6O | 0.27 | 77.03883; 51.02321; 107.04957; 50.01562 |
| 45 | Schisandrin B | 15.939 | 401.19386 | [M+H]+ | C23H28O6 | -5 | 300.09799; 285.07452; 301.10570; 270.08758 |
| 46 | Schisandrin C | 16.146 | 385.16437 | [M+H]+ | C22H24O6 | -0.51 | 285.07583; 227.07059; 257.08146; 255.06586 |
| 47 | L-Asparagine | 0.907 | 131.04648 | [M-H]- | C4H8N2O3 | -0.18 | 72.00864; 70.03035; 113.03615; 58.02961 |
| 48 | L-Aspartic acid | 0.915 | 132.03038 | [M-H]- | C4H7NO4 | -1.51 | 97.93170; 102.97068; 58.98410; 86.97749 |
| 49 | Mucic acid | 0.933 | 209.03082 | [M-H]- | C6H10O8 | -0.24 | 115.07684; 129.05600; 72.99327; 143.07175 |
| 50 | Bergapten | 0.944 | 215.03304 | [M-H]- | C12H8O4 | -0.81 | 99.92517; 134.89441; 136.89259; 210.82125 |
| 51 | L-Tartaric acid | 1.007 | 149.0094 | [M-H]- | C4H6O6 | 2.02 | 145.89078; 58.98101; 102.94868; 146.90008 |
| 52 | D-Malic acid | 1.148 | 133.01436 | [M-H]- | C4H6O5 | 1.13 | 99.92573; 115.92088; 100.93328; 83.93088 |
| 53 | Citramalic acid | 1.239 | 147.03007 | [M-H]- | C5H8O5 | 2.53 | 87.00899; 57.03486; 85.02993; 59.01393 |
| 54 | Neochlorogenic acid | 4.724 | 353.0878 | [M-H]- | C16H18O9 | -9.03 | 163.11283; 177.09215; 353.21239; 190.09995 |
| 55 | 3-p-Coumaroylquinic acid | 5.496 | 337.09274 | [M-H]- | C16H18O8 | 1.6 | 322.08528; 307.06082; 278.05836; 337.10846 |
| 56 | Cryptochlorogenic acid | 5.702 | 353.08779 | [M-H]- | C16H18O9 | 0.85 | 353.19946; 96.95996; 116.92744; 79.95592 |
| 57 | 3-Feruloylquinic acid | 5.874 | 367.10313 | [M-H]- | C17H20O9 | 1.18 | 119.04997; 369.13319; 93.29779; 119.06452 |
| 58 | 8-Arabinosyl-6-glucosylluteolin | 6.179 | 579.13647 | [M-H]- | C26H28O15 | -0.02 | 579.13630; 369.06198; 399.07276; 489.10432 |
| 59 | Sulfo jasmonate | 6.211 | 305.06971 | [M-H]- | C12H18O7S | -0.45 | 142.06604; 304.14588; 304.18933; 131.06073 |
| 60 | Taxifolin 7-O-glucoside | 6.279 | 449.10853 | [M-H]- | C21H22O11 | -0.04 | 119.04863; 299.16971; 443.11326; 461.12195 |
| 61 | Quinic acid | 6.368 | 191.05577 | [M-H]- | C7H12O6 | -0.89 | 172.05158; 187.07454 |
| 62 | NP-000062(6) | 6.452 | 563.14106 | [M-H]- | C26H28O14 | 1.6 | 617.38524; 145.02857; 617.43941; 618.38777 |
| 63 | D-(-)-Quinic acid | 6.629 | 191.056 | [M-H]- | C7H12O6 | -0.9 | 172.05161; 157.06450; 185.06189; 187.07575 |
| 64 | 3-O-Feruloylquinic acid | 6.631 | 367.10321 | [M-H]- | C17H20O9 | -1.79 | 116.92854; 119.05034; 59.01389; 99.92590 |
| 65 | Quercetin 3-O-xylosylglucoside | 6.663 | 595.13132 | [M-H]- | C26H28O16 | 0.77 | 300.02744; 595.13019; 271.02478; 301.03467 |
| 66 | Neoeriocitrin | 6.864 | 595.16776 | [M-H]- | C27H32O15 | -0.58 | 385.92383; 339.91952; 429.91450; 313.94134 |
| 67 | Rutin | 6.884 | 609.147 | [M-H]- | C27H30O16 | -0.67 | 687.22462; 119.05104; 687.25827; 509.12785 |
| 68 | 3-Hydroxysuberic acid | 6.888 | 189.07685 | [M-H]- | C8H14O5 | 1.45 | 129.05561; 99.08177; 127.07653; 57.03472 |
| 69 | Isovitexin | 7.011 | 431.09864 | [M-H]- | C21H20O10 | 1.54 | 145.02945; 128.03570; 164.07255; 275.10397 |
| 70 | Isorhamnetin 3-O-xylosylglucoside | 7.066 | 609.14594 | [M-H]- | C27H30O16 | 1.46 | 314.04352; 609.14606; 299.01994; 315.05132 |
| 71 | Isoquercetin | 7.089 | 463.08847 | [M-H]- | C21H20O12 | 0.02 | 111.00871; 256.91196; 479.10224; 67.01865 |
| 72 | Narcissin | 7.333 | 623.16202 | [M-H]- | C28H32O16 | 0.63 | 723.25214; 111.00827; 173.00905; 723.29584 |
| 73 | Astragalin | 7.507 | 447.09286 | [M-H]- | C21H20O11 | -0.28 | 237.94827; 118.89969; 458.02092; 422.04545 |
| 74 | Umbelliferone | 7.536 | 161.0242 | [M-H]- | C9H6O3 | 0.58 | 161.02474; 133.03011; 105.03473; 77.03986 |
| 75 | Diosmin | 7.536 | 607.16743 | [M-H]- | C28H32O15 | 0.42 | 299.05612; 284.03229; 607.16595; 607.10732 |
| 76 | 22,24,29-Trihydroxy-29-oxooleanane glycoside | 9.65 | 971.48496 | [M-H]- | C48H76O20 | -1.35 | 971.48450; 972.48992; 971.55444; 645.36406 |
| 77 | Corchorifatty acid F | 10.102 | 327.21767 | [M-H]- | C18H32O5 | 0.97 | 327.26634; 145.09908; 283.28255; 171.10120 |
| 78 | Coumestrol | 10.121 | 267.02972 | [M-H]- | C15H8O5 | -0.78 | 147.04489; 119.05021; 218.11824; 93.03534 |
| 79 | 8-Prenylnaringenin | 10.326 | 339.12315 | [M-H]- | C20H20O5 | -0.08 | 119.05021; 219.06611; 133.06589; 339.12375 |
| 80 | (9Z)-5,8,11-Trihydroxyoctadec-9-enoic acid | 10.339 | 329.23343 | [M-H]- | C18H34O5 | -0.66 | 329.23401; 211.13463; 171.10323; 229.14510 |
| 81 | Glycyrrhizin | 10.774 | 821.39687 | [M-H]- | C42H62O16 | -1.91 | 821.39505; 351.05621; 822.39699; 193.03453 |
| 82 | Isobavachin | 11.252 | 323.12894 | [M-H]- | C20H20O4 | 0.44 | 211.03887; 265.04767; 267.02834; 293.07959 |
| 83 | Evodol | 11.37 | 483.16615 | [M-H]- | C26H28O9 | 0.18 | 421.16547; 161.06074; 423.14544; 395.15071 |
| 84 | Neobavaisoflavone | 11.579 | 321.11265 | [M-H]- | C20H18O4 | 0.19 | 321.11311; 265.05043; 277.05071; 195.04496 |
| 85 | (9Z,12E)-15,16-Dihydroxyoctadeca-9,12-dienoic acid | 11.904 | 311.22289 | [M-H]- | C18H32O4 | -1.81 | 249.22278; 293.21396; 119.05030; 311.22379 |
| 86 | Evodiamine | 11.972 | 302.12993 | [M-H]- | C19H17N3O | 0.34 | 108.02147; 286.12039; 136.01658; 203.03467 |
| 87 | Desmethylxanthohumol | 13.013 | 339.12372 | [M-H]- | C20H20O5 | 0.15 | 119.05043; 219.06647; 133.06608; 339.12397 |
| 88 | Ceanothic acid | 13.621 | 485.32732 | [M-H]- | C30H46O5 | -0.23 | 485.32707; 423.32733; 485.29168; 485.40229 |
| 89 | 9-HOTrE | 13.636 | 293.21217 | [M-H]- | C18H30O3 | 0.15 | 275.20095; 183.13843; 293.21230; 235.16995 |
| 90 | Corylifol A | 14.045 | 389.17543 | [M-H]- | C25H26O4 | -0.16 | 208.05328; 135.00918; 209.06127; 145.02985 |
| 91 | 9-HODE | 14.258 | 295.22781 | [M-H]- | C18H32O3 | -1.04 | 267.02896; 295.02259; 266.02027; 122.03783 |
| 92 | Bakuchiol | 15.892 | 255.17539 | [M-H]- | C18H24O | -0.2 | 94.02943; 165.95908; 161.88489; 163.98126 |

**Table S2. Primer sequences used for RT-qPCR**

| **Gene** | **Forward primer（5′–3′）** | **Reverse primer（5′–3′）** |
| --- | --- | --- |
| Egr2 | CCTTTGACCAGATGAACGGAGTG | CTGGTTTCTAGGTGCAGAGATGG |
| Rgs4 | AGAAATGGGCTGAATCGTTG | GGCTTGTCTCCTCTCTGGTG |
| Cdkn1a | TCGCTGTCTTGCACTCTGGTGT | CCAATCTGCGCTTGGAGTGATAG |
| Hprt1 | CTGGTGAAAAGGACCTCTCGAAG | CCAGTTTCACTAATGACACAAACG |

**Table S3. Effect of Sishen Wan on brain structure in mice with depression induced by chronic sleep**

| **Contrast** | **Brain region** | | **MNI coordinates** | | | **Voxel**  **size** | | **T-Value** |
| --- | --- | --- | --- | --- | --- | --- | --- | --- |
| **X** | | | | **Y** | **Z** | | | |
| **Model>Control** Hippocampus_left | | - 1.4151 | | 0.9344 | -2.7402 | | 6144 | 2.8882 |
| Hippocampus_right | | 1.8903 | | 4.4339 | -3.8785 | | 5783 | 3.2492 |
| Subiculum_left | | - 1.4151 | | 0.9344 | -2.7402 | | 1457 | 2.8882 |
| Subiculum_right | | 2.4657 | | 3.6437 | -4.7127 | | 1373 | 2.9311 |
| Amygdala_left | | -3.2319 | | 4.2171 | -2.1076 | | 209 | 2.4047 |
| Amygdala_right | | 1.9857 | | 4.3593 | - 1.8804 | | 118 | 2.2357 |
| **Model>SSW** Hippocampus_left | | -2.2671 | | 3.8762 | -3.8777 | | 8181 | -3.4829 |
| Hippocampus_right | | 2.0829 | | 4.343 | -3.6448 | | 7006 | -4.8599 |
| Subiculum_left | | 3.2265 | | 5.0477 | -3.626 | | 2612 | -3.9257 |
| Subiculum_right | | 2.9559 | | 4.9675 | -3.4034 | | 3192 | -3.7932 |
| Amygdala_left | | - 1.8771 | | 4.6267 | - 1.8698 | | 2205 | 3.5331 |
| Amygdala_right | | 2.8605 | | 5.1843 | -3.2829 | | 2656 | -3.6502 |

**Table S4. Effect of Sishen Wan on the amplitude of low-frequency fluctuations in mice with**

**depression induced by chronic sleep deprivation**

| **Contrast** | **Brain region** | | **MNI coordinates** | | | **Voxel size** | | **T-Value** |
| --- | --- | --- | --- | --- | --- | --- | --- | --- |
| **X** | | | | **Y** | **Z** | | | |
| **Model＞Control** | Hippocampus_left | - 1.9773 | | 3.9428 | -2.819 | | 3520 | 3.936 |
|  | Hippocampus_right | 3.0387 | | 2.652 | -2.3742 | | 7118 | 6.3131 |
|  | Subiculum_left | -2.0853 | | 1.8555 | -4.609 | | 618 | 3.2231 |
|  | Subiculum_right | 2.3607 | | 2.2566 | -3.7891 | | 2005 | 4.9233 |
|  | Amygdala_left | - 1.9749 | | 4.4129 | - 1.9899 | | 912 | 3.4298 |
|  | Amygdala_right | 2.8575 | | 4.7053 | -2.7009 | | 1145 | 3.5039 |
| **Model＞SSW** | Hippocampus_left | -2.6493 | | 4.6002 | -3.9855 | | 6399 | -5.7358 |
|  | Hippocampus_right | 2.4711 | | 4.6639 | -3.4063 | | 9426 | -8.1125 |
|  | Subiculum_left | -2.6493 | | 4.5923 | -4.1032 | | 1500 | -5.8122 |
|  | Subiculum_right | 2.8569 | | 4.5226 | -3.8792 | | 2961 | 7.3097 |
|  | Amygdala_left | - 1.8735 | | 5.3753 | 0.0212 | | 1110 | -4.908 |
|  | Amygdala_right | 2.5647 | | 4.2229 | -2.236 | | 2611 | -4.6896 |

**Table S5. Effect of Sishen Wan on regional homogeneity in mice with depression induced by chronic sleep deprivation**

| **Contrast** | **Brain region** | | **MNI coordinates** | | | **Voxel size** | | **T-Value** |
| --- | --- | --- | --- | --- | --- | --- | --- | --- |
| **X** | | | | **Y** | **Z** | | | |
| **Model>**  **Control** | Hippocampus_left | - 1.6827 | | 4.7363 | -3.28 | | 1841 | 4.8344 |
|  | Hippocampus_right | 2.7519 | | 3.0802 | -3.7795 | | 3591 | 4.3685 |
|  | Subiculum_left | -2.1621 | | 5.3581 | -3.3889 | | 331 | 4.3356 |
|  | Subiculum_right | 3.1359 | | 2.6199 | -4.374 | | 858 | 5.6081 |
|  | Amygdala_left | 1.9695 | | 5.4805 | 0.0227 | | 958 | 3.8422 |
|  | Amygdala_right | 3.2391 | | 3.9013 | -2.2413 | | 202 | 2.8913 |
| **Model>SSW** | Hippocampus_left | -2.7477 | | 4.3222 | -3.5184 | | 2549 | -5.6403 |
|  | Hippocampus_right | 3.5241 | | 3.0356 | -2.7231 | | 4910 | -9.6945 |
|  | Subiculum_left | -3.0375 | | 4.2872 | -4.1063 | | 598 | -5.8213 |
|  | Subiculum_right | 3.1455 | | 4.3028 | -4.0001 | | 1686 | -6.3947 |
|  | Amygdala_left | -2.1597 | | 5.9862 | -0.2058 | | 711 | -4.3511 |
|  | Amygdala_right | 1.1229 | | 5.6399 | 0.0189 | | 587 | -3.1974 |

**Table S6. Differential serum metabolites identified in each group under positive- and negative-ion modes**

| **NO.** | | **HMDB** | **TR**  **（min）** | | | **m/z** | **Mass**  **Error** | | | **Identification** | **Adduct**  **type** | | | **Formula** | **Control**  **VS**  **Model** | | **Model VS**  **SSW** | | |
| --- | --- | --- | --- | --- | --- | --- | --- | --- | --- | --- | --- | --- | --- | --- | --- | --- | --- | --- | --- |
| 1 | | HMDB0040701 | 5.93 | | | 371.2075 | -0.003 | | | 9-Hydroxy-7-megastigmen-3-one  glucoside | M-H | | | C19H32O7 | ↑** | | ↓ | | |
| 2 | | HMDB0005368 | 3.68 | | | 887.8075 | 0.19 | | | TG(16:0/18:0/20:1(11Z)) | M-H | | | C57H108O6 | ↓* | | ↑## | | |
| 3 | | HMDB0030124 | 4.09 | | | 131.0714 | -0.12 | | | Prenol | M+FA-H | | | C5H10O | ↓** | | ↑## | | |
| 4 | | HMDB0011518 | 11 | | | 546.2819 | -3.66 | | | LysoPE(20:4(8Z,11Z,14Z,17Z)/0:0) | M+FA-H | | | C25H44NO7P | ↑** | | ↓ | | |
| 5 | | HMDB0010395 | 7.15 | | | 588.3309 | 0.33 | | | LysoPC(20:4(5Z,8Z,11Z,14Z)/0:0) | M+FA-H | | | C28H50NO7P | ↑** | | ↓ | | |
| 6 | | HMDB0011473 | 7.26 | | | 452.2773 | -2.12 | | | LysoPE(0:0/16:0) | M-H | | | C21H44NO7P | ↑** | | ↓ | | |
| 7 | | HMDB0115952 | 8.28 | | | 974.4903 | - 1.03 | | | CDP-G(16:1(9Z)/18:2(9Z,12Z)) | M-H | | | C46H79N3O15P2 | ↓** | | ↑# | | |
| 8 | | HMDB0283362 | 8.62 | | | 948.4898 | 2.05 | | | PS(22:5(4Z,7Z,10Z,13Z,16Z)/20:5(7Z,  9Z,11E,13E,17Z)-3OH(5,6,15)) | M+FA-H | | | C48H74NO13P | ↓** | | ↑# | | |
| 9 | | HMDB0285366 | 8.72 | | | 800.5431 | - 1.99 | | | PE(P-18:0/PGE1) | M-H | | | C43H80NO10P | ↓** | | ↑## | | |
| 10 | | HMDB0008107 | 8.72 | | | 826.5587 | -2.10 | | | PC(18:1(9Z)/18:3(9Z,12Z,15Z)) | M+FA-H | | | C44H80NO8P | ↓** | | ↑ | | |
| 11 | | HMDB0283850 | 8.72 | | | 850.5586 | -2.11 | | | PE(22:1(13Z)/PGJ2) | M-H | | | C47H82NO10P | ↓** | | ↑## | | |
| 12 | | HMDB0283990 | 8.72 | | | 874.5645 | 4.79 | | | PE(22:2(13Z,16Z)/22:6(5Z,8E,10Z,13Z  ,15E,19Z)-2OH(7S, 17S)) | M-H | | | C49H82NO10P | ↓** | | ↑# | | |
| 13 | | HMDB0007074 | 9.10 | | | 577.4828 | - 1.69 | | | DG(15:0/18:2(9Z,12Z)/0:0) | M-H | | | C36H66O5 | ↓** | | ↑# | | |
| 14 | | HMDB0038061 | 9.61 | | | 977.7692 | 3.15 | | | Triricinolein | M+FA-H | | | C57H104O9 | ↓** | | ↓# | | |
| 15 | | HMDB0012225 | 1.36 | | | 165.0544 | - 1.51 | | | Enol-phenylpyruvate | M+H | | | C9H8O3 | ↓** | | ↑# | | |
| 16 | | HMDB0007980 | 9.03 | | | 806.5707 | 4.72 | | | PC(16:0/20:3(5Z,8Z,11Z) | M+Na | | | C44H82NO8P | ↓* | | ↑## | | |
| 17 | | HMDB0011617 | 0.68 | | | 348.0700 | -1.02 | | | Adenosine 2'-phosphate | M+H,M+N | | | C10H14N5O7P | ↓** | | ↑# | | |
| 18 | HMDB0031641 | | | 3.68 | 72.0807 | | | -0.26 | Pyrrolidine | | | M+H | C4H9N | | | ↓** | | ↑## |  |
| 19 | HMDB0000696 | | | 0.85 | 150.0581 | | | - 1.31 | L-Methionine | | | M+H | C5H11NO2S | | | ↓** | | ↑## |  |
| 20 | HMDB0250803 | | | 1.36 | 182.0809 | | | - 1.45 | D-Tyrosine | | | M+H | C9H11NO3 | | | ↓** | | ↑ |  |
| 21 | HMDB0001645 | | | 4.16 | 132.1015 | | | -2.91 | L-Norleucine | | | M+H | C6H13NO2 | | | ↓** | | ↑## |  |
| 22 | HMDB0001007 | | | 4.33 | 166.0862 | | | -0.37 | 3-Pyridinebutanoic acid | | | M+H | C9H11NO2 | | | ↓** | | ↑# |  |
| 23 | HMDB0000222 | | | 6.68 | 400.3432 | | | 2.62 | Palmitoylcarnitine | | | M+H | C23H45NO4 | | | ↑** | | ↓ |  |
| 24 | HMDB0005065 | | | 6.81 | 426.3581 | | | 0.76 | Oleoylcarnitine | | | M+H | C25H47NO4 | | | ↑** | | ↓ |  |
| 25 | HMDB0011496 | | | 6.98 | 526.2936 | | | 1.49 | LysoPE(0:0/22:6(4Z,7Z,10Z,13Z,16Z,1  9Z)) | | | M+H | C27H44NO7P | | | ↑** | | ↓ |  |
| 26 | HMDB0031371 | | | 7.06 | 1093.6198 | | | 4.08 | Notoginsenoside I | | | M+H | C54H92O22 | | | ↑** | | ↓ |  |
| 27 | HMDB0287901 | | | 7.09 | 1021.6282 | | | -0.45 | PC(22:1(13Z)/LTE4) | | | M+Na | C53H95N2O11PS | | | ↑* | | ↓ |  |
| 28 | HMDB0004249 | | | 8.96 | 644.9772 | | | - 1.54 | Phosphatidylinositol-3,4,5- trisphosphate | | | M+H | C12H24O22P4 | | | ↑** | | ↓# |  |
| 29 | HMDB0289429 | | | 9.64 | 804.5542 | | | 3.59 | PC(P-16:0/20:4(5Z,8Z,11Z,14Z)-  OH(20))  PC(P- | | | M+Na | C44H80NO8P | | | ↓** | | ↑## |  |
| 30 | HMDB0289501 | | | 9.64 | 828.5530 | | | 2.05 | 16:0/22:6(5Z,7Z,10Z,13Z,16Z,19Z)-  OH(4)) | | | M+Na | C46H80NO8P | | | ↓** | | ↑## |  |
| 31 | HMDB0000024 | | | 9.95 | 892.6522 | | | -2.29 | 3-O-Sulfogalactosylceramide  (d18:1/24:0) | | | M+H | C48H93NO11S | | | ↑** | | ↓ |  |

**Figure S1. UPLC–QTOF–MS total ion chromatograms of Sishen Wan in positive and negative ion modes.** A, representative total ion chromatogram obtained in positive ion mode; B, representative total ion chromatogram obtained in negative ion mode. Peak numbers correspond to the putatively annotated constituents listed in Table S1.

**Figure S2. OPLS-DA score chart and permutation test of different groups in positive and negative ion modes.** A, OPLS-DA score plots for the Control vs. Model and Model vs. SSW comparisons. B, Corresponding 100-permutation tests validating the robustness of the OPLS-DA models.
